# Identification of metabolism pathways directly regulated by sigma^54^ factor in *Bacillus thuringiensis*

**DOI:** 10.3389/fmicb.2015.00407

**Published:** 2015-05-12

**Authors:** Qi Peng, Guannan Wang, Guiming Liu, Jie Zhang, Fuping Song

**Affiliations:** ^1^State Key Laboratory for Biology of Plant Diseases and Insect Pests, Institute of Plant Protection, Chinese Academy of Agricultural SciencesBeijing, China; ^2^CAS Key Laboratory of Genome Sciences and Information, Beijing Institute of Genomics, Chinese Academy of SciencesBeijing, China

**Keywords:** *Bacillus thuringiensis*, sigma^54^, bEBPs, metabolic pathways, DNA microarray

## Abstract

Sigma^54^ (σ^54^) regulates nitrogen and carbon utilization in bacteria. Promoters that are σ^54^-dependent are highly conserved and contain short sequences located at the −24 and −12 positions upstream of the transcription initiation site. σ^54^ requires regulatory proteins known as bacterial enhancer-binding proteins (bEBPs) to activate gene transcription. We show that σ^54^ regulates the capacity to grow on various nitrogen sources using a *Bacillus thuringiensis* HD73 mutant lacking the *sigL* gene encoding σ^54^ (Δ*sigL*). A 2-fold-change cutoff and a false discovery rate cutoff of *P* < 0.05 were used to analyze the DNA microarray data, which revealed 255 genes that were downregulated and 121 that were upregulated in the Δ*sigL* mutant relative to the wild-type HD73 strain. The σ^54^ regulon (stationary phase) was characterized by DNA microarray, bioinformatics, and functional assay; 16 operons containing 47 genes were identified whose promoter regions contain the conserved −12/−24 element and whose transcriptional activities were abolished or reduced in the Δ*sigL* mutant. Eight σ^54^-dependent transcriptional bEBPs were found in the Bt HD73 genome, and they regulated nine σ^54^-dependent promoters. The metabolic pathways activated by σ^54^ in this process have yet to be identified in *Bacillus thuringiensis*; nonetheless, the present analysis of the σ^54^ regulon provides a better understanding of the physiological roles of σ factors in bacteria.

## Introduction

Sigma (σ) factors are a class of proteins constituting dissociable subunits of prokaryotic RNA polymerase. Different σ factors associate with the core polymerase and recognize the promoters of target genes to initiate transcription in response to environmental conditions. Identifying regulons in bacteria that are controlled by specific σ factors is important for understanding cell responses to given stimuli.

The *sigL* gene (known as *rpoN* gene in Gram-negative soil bacteria) encodes σ^54^. Promoters that are σ^54^-dependent are highly conserved and contain short sequences located at the −24 and −12 positions upstream of the transcription initiation site, in contrast to σ^70^-dependent promoters containing upstream sequences located at −35 and −10 (Buck et al., 2000). As observed for eukaryotic enhancer-binding proteins (EBPs), σ^54^ requires regulatory proteins known as bacterial (b)EBPs to activate gene transcription (Bush and Dixon, 2012).

σ^54^ activates genes that are involved in the utilization of nitrogen and carbon for energy and a range of other cellular processes in bacteria (Merrick, 1993). The Sigma 54 regulon is found in several species, including *Escherichia coli* (Reitzer and Schneider, 2001), *Pseudomonas putida* (Cases et al., 2003), *Vibrio cholera* (Dong and Mekalanos, 2012), *Xylella fastidiosa* (da Silva Neto et al., 2010), *Geobacter sulfurreducens* (Leang et al., 2009)*, Listeria monocytogenes* (Arous et al., 2004), and several members of Rhizobiaceae family (Dombrecht et al., 2002; Hauser et al., 2007). *E. coli* has approximately 30 σ^54^-dependent operons, about half of which are involved in nitrogen assimilation and metabolism; the others participate in the conservation of metabolites and energy resources under adverse conditions (Reitzer and Schneider, 2001), and are related to arginine and histidine transport (Caldara et al., 2007), arginine catabolism (Kiupakis and Reitzer, 2002), acetoacetate catabolism (Matta et al., 2007), nitrogen assimilation (Magasanik, 1989; Yamada et al., 2007), glutamine transport (Baev et al., 2006), and propionate catabolism (Lee et al., 2005). In *V. cholera*, 68 σ^54^-binding sites and 82 operons positively regulated by σ^54^ have been identified, of which 37 binding sites are confirmed by chromatin immunoprecipitation (Dong and Mekalanos, 2012).

Among Gram-positive bacteria, 77 genes have been identified in *L. monocytogenes*, most of which are related to carbohydrate (e.g., pyruvate) metabolism (Arous et al., 2004). Several metabolism-related operons regulated by σ^54^ have also been identified in *Bacillus subtilis* such as those involved in isoleucine and valine utilization (Debarbouille et al., 1999), the acetoin (3-hydroxy 2-butanone) catabolic pathway (Ali et al., 2001), and arginine catabolism (Heidrich et al., 2006), as well as in the degradation of polymers of fructose (levanes) (Debarbouille et al., 1991a).

*B. anthracis*, *B. cereus*, and *B. thuringiensis* (Bt) are all spore-forming members of the *B. cereus* group (Helgason et al., 2000). These species vary in terms of host range and virulence (Han et al., 2006), and are mainly distinguished by the genes contained in their plasmids. Bt forms parasporal crystals during the stationary phase of growth that are toxic to a wide variety of insect larvae (Schnepf et al., 1998), making Bt strains the most commonly used biological pesticide worldwide. Among these species, only two metabolic pathways are known to be controlled by σ^54^: the γ-aminobutyric acid (GABA) (Zhu et al., 2010) and l-lysine metabolism (Zhang et al., 2014) pathways in the Bt HD73 strain. Little is known about the roles of σ^54^ and its regulons in the *B. cereus* group. Thus, the aim of the present study was to investigate these roles using the HD73 strain of Bt.

Regulons and binding sites of σ^54^ were identified by DNA microarray and computational predictions, which were also used to analyze EBP domains and gene organization and predict operons. The results revealed several novel σ^54^-dependent metabolic pathways. These findings provide insight into σ^54^-dependent regulation in the *B. cereus* group and demonstrate that the σ^54^ regulon in Bt differs from those of other Gram-positive bacteria.

## Materials and methods

### Bacterial strains, plasmids, and culture

Bacterial strains and plasmids used in this study are listed in Additional file 4. *E. coli* TG1 was used for cloning, while ET12567 was used to produce unmethylated plasmid DNA for Bt transformation (Wang et al., 2006). Wild-type Bt HD73 (laboratory stock) expressing *cry1Ac* was used throughout this study. Bt strains were transformed by electroporation as previously described (Wang et al., 2006). *E. coli* was grown at 37°C in Luria-Bertani (LB) medium (1% NaCl, 1% tryptone, and 0.5% yeast extract). Bt was grown in LB medium, Schaeffer's sporulation medium (SSM) (Schaeffer et al., 1965), or glucose minimal medium (GMM) (Debarbouille et al., 1991b) supplemented with 40 mM of a given amino acid as the sole nitrogen source, with vigorous shaking (220 rpm) at 30°C. The antibiotic concentrations used for bacterial selection were as follows: 100 μg/ml ampicillin for *E. coli*, 5 μg/ml erythromycin and 50 μg/ml kanamycin for Bt. Bacteria producing β-galactosidase were identified by culturing in SSM medium.

### DNA manipulation techniques

Plasmid DNA was extracted from *E. coli* by a standard alkaline lysis procedure with a plasmid miniprep kit (Axygen Scientific, Union City, CA, USA). Chromosomal DNA was extracted from Bt cells as previously described (Lereclus et al., 1989). Restriction enzymes and T4 DNA ligase were used according to the manufacturer's instructions (New England Biolabs, Ipswich, MA, USA). Oligonucleotide primers were synthesized by Sangon (Shanghai, China); all primer sequences are listed in Additional file 5. PCR was performed with high-fidelity DNA polymerase (Toyobo, Osaka, Japan). Amplified fragments were purified with a PCR cleanup kit (Axygen). Digested DNA fragments were separated on 1% agarose gels and extracted using a DNA gel extraction kit (Axygen). All constructs were confirmed by sequencing (Invitrogen, Carlsbad, CA, USA).

### Preparation of RNA for DNA microarray

Total RNA was extracted from Bt cells grown in SSM medium at stage T_7_ (7 h after the end of the exponential phase). Three independent repeats from different clones were performed for each strain. Cells were harvested from 1 ml of culture by centrifugation (13,000 × *g* for 30 s at 4°C), and cell pellets were immediately suspended in 1 ml cold TRI-Reagent (Invitrogen). The suspensions were then snap-frozen in liquid nitrogen. The RNA was extracted using the Qiagen Easy RNA kit (Hilden, Germany). Residual DNA was removed using RNase-free DNase I (New England Biolabs), and the resulting RNA samples were stored at −80°C.

### RNA amplification, labeling, and hybridization

Total RNA was amplified and labeled with the Low Input Quick Amp Labeling Kit, One-Color (Agilent Technologies, Santa Clara, CA, USA). Labeled RNA was purified using the RNeasy mini kit (Qiagen). Each slide was hybridized with 1.65 μg Cy3-labeled cRNA using the Gene Expression Hybridization Kit in a hybridization oven (both from Agilent Technologies). After 17 h, the slides were washed in staining dishes (Thermo Fisher Scientific, Waltham, MA, USA) using the Gene Expression Wash Buffer kit (Agilent Technologies).

### Data acquisition

Slides were scanned using a Microarray Scanner (Agilent Technologies) with default settings (dye channel: green; scan resolution: 5 μm; photomultiplier tube: 100%; 10%, 16-bit) and Feature Extraction software 10.7 (Agilent Technologies). Raw data were normalized with the quantile algorithm of Gene Spring Software 11.0 (Agilent Technologies). Transcriptome data were analyzed with statistical tests.

### Construction of −12/−24 promoter with *lacZ* fusions

The predicted −12/−24 regions of promoters were amplified from Bt HD73 genomic DNA using specific primers. Promoter restriction fragments were then ligated into the pHT304-18Z vector containing a promoterless *lacZ* gene (Agaisse and Lereclus, 1994). Recombinant pHT-Pn (where n indicates the gene ID in the Bt HD73 genome) was introduced into Bt HD73 and Δ*sigL* mutant strains. The resultant strains, HD73(Pn) and Δ*sigL*(Pn), were selected by resistance to erythromycin and tested by PCR to confirm the presence of the promoter fragments in the plasmids.

### β-galactosidase assay

Bt strains were cultured in SSM medium at 30°C and 220 rpm. A 2-ml volume was collected at 1-h intervals from T_1_ to T_8_, from which cells were harvested by centrifugation for 1 min at 10,000 × *g*. The supernatant was removed, and the pellet was stored at −20°C or resuspended in 500 μl Buffer Z with 1 mM dithiothreitol. The β-galactosidase activity was determined as previously described (Perchat et al., 2011) and expressed as Miller units. Reported values represent averages from at least three independent assays.

### Construction of EBP mutants

Five EBP (*rocR*, *prdR*, *acoR*, *bkdR*, and *levR*) deletion strains were generated by allelic exchange as previously described (Zhu et al., 2010) using a kanamycin resistance cassette (*kan*, 1473 bp) and the thermosensitive suicide plasmid pMAD (Arnaud et al., 2004). The upstream and downstream regions (fragments A and B, respectively) of the *hd_0559* (*rocR*) gene were amplified by PCR using chromosomal DNA from Bt as template and rocR-AF/rocR-AR and rocR-BF/rocR-BR primers. The *kan* gene was amplified by PCR using pDG780 (Guerout-Fleury et al., 1995) as template and rocR-kmF/rocR-kmR primers. Fragment A and *kan* were ligated together by overlapping PCR using primers rocR-AF and rocR-kmR. The amplification product was then integrated with fragment B in a second round of overlapping PCR using primers rocR-AF and rocR-BR. The resultant PCR products were digested, purified, and ligated with the pMAD plasmid to generate pMADΔrocR. The host strain was transformed with the recombinant plasmid by electroporation, and erythromycin-sensitive transformants were selected. The correct mutant strain was identified by PCR. The *hd_1069* (*prdR*), *hd_3228* (*acoR*), *hd_4469* (*bkdR*), and *hd_5607* (*levR*) deletion strains were constructed as described above.

The *hd_3141* (*soxR*) deletion mutant was constructed as follows. DNA fragments corresponding to upstream and downstream regions of the *soxR* gene were amplified by PCR using genomic DNA from Bt HD73 as the template and the *soxR*-a/*soxR*-d and *soxR*-b/*soxR*-c primers. The amplified fragments were fused via overlapping PCR using the *soxR*-a/*soxR*-b primers. The resultant 1257-bp fragment was then digested with the *Bam*HI and *Eco*RI restriction enzymes and inserted between the corresponding restriction sites of the pMAD plasmid. The recombinant pMADΔsoxR plasmid was electroporated into host strains and erythromycin-sensitive transformants were selected.

## Results

### Characterization of the *sigL* mutant of Bt

Individual l-amino acids were tested for their ability to support the growth of HD73 and Δ*sigL* mutant strains in a GMM. These two strains were unable to use alanine, aspartate, asparagine, glutamic acid, glutamine, threonine, cysteine, phenylalanine, tyrosine, glycine, lysine, and methionine as nitrogen sources (data not shown), but were able to use histidine and arginine (Table 1). In addition, the Δ*sigL* mutant was unable to use sarcosine, leucine, isoleucine, serine, or valine as nitrogen sources, unlike the HD73 strain (Table 1). These results suggest that σ^54^ controls the pathways responsible for the utilization of these amino acids.

**Table 1:** **Doubling time of HD73 wild type and Δ*sigL* mutant strains grown in minimal medium containing various nitrogen sources**.

**Nitrogen source**	**Doubling time (hour)**	
	**HD73**	**ΔsigL**
Sarcosine	16.54	>60
Proline	30.13	>60
Histidine	15.93	16.91
Leucine	18.65	>60
Isoleucine	10.69	>60
Serine	16.84	>60
Arginine	9.71	8.75
Valine	14.74	>60

### Functional classification of differentially expressed genes in wild-type and Δ*sigL* mutant strains

To further investigate the function of σ^54^ in Bt, the transcriptome of HD73 and Δ*sigL* mutant strains was compared in order to identify potential transcriptional targets of σ^54^. A 2-fold-change cutoff and a false discovery rate cutoff of *P* < 0.05 were used to analyze the DNA microarray data, which revealed 255 genes that were downregulated and 121 that were upregulated in the Δ*sigL* mutant relative to the wild-type HD73 strain. DNA microarray data were deposited at the NCBI Gene Expression Omnibus (accession no. GSE48410). A complete list of the differentially expressed genes and their expression ratios is provided as Additional file 1. The genes were assigned to five functional groups according to Kyoto Encyclopedia of Genes and Genomes, Clusters of Orthologous Groups, and Pfam protein family classifications of the Bt subsp. *kurstaki* strain HD73 genome, most encoding hypothetical proteins or those of unknown function proteins (117 and 72 that were down- and upregulated, respectively). The top three categories comprised genes associated with amino acid metabolism (66 down- and five upregulated), carbohydrate metabolism (17 down- and 15 upregulated) and intracellular transport system (29 down- and 11 upregulated). Other genes were associated with sporulation (17 down- and two upregulated) and signal transduction (nine down- and 16 upregulated). More than 70% of the genes were associated with amino acid and carbohydrate metabolism.

### Validation of predicted σ^54^-related promoter elements with transcriptional *lacZ* fusion constructs

A computational analysis was carried out to identify σ^54^ recognition sequences within the HD73 genome. The conserved sequence BYGGCMYRNNNNYYGCW (Francke et al., 2011) was searched 700 bp upstream and 100 bp downstream of start codons. After eliminating the coding regions, 14 putative σ^54^-binding sites were found in the same strand orientation as their potential target genes (Additional file 2). Of these, 10 were located in genes that were downregulated in the DNA microarray data and one was in an upregulated gene, which was fewer than expected. To identify genes potentially controlled by σ^54^ from an expanded pool, a search for the minimally conserved sequence NNGGN_10_GCNN (critical bases underlined) was carried out in the region 700 bp upstream of the start codon of genes that were downregulated in the DNA microarray. Using this approach, 17 promoters were identified containing the target sequence, including the 10 putative σ^54^-binding sites previously predicted with the highly conserved sequence, for a total of 21 genes potentially regulated by σ^54^ (Additional file 2).

**Figure 1:**
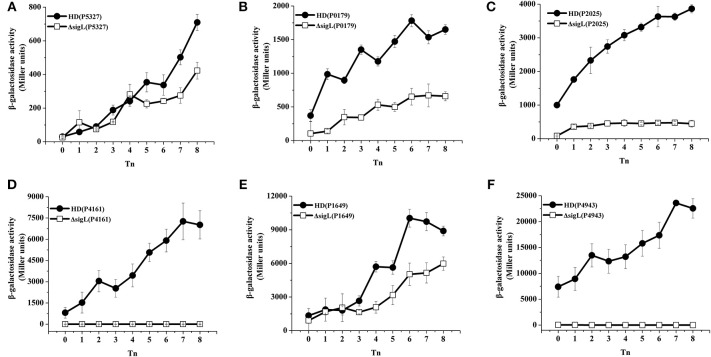
**β-galactosidase activity assay of the putative −12/−24 promoters**. Wild-type strain HD73 (•) and the *sigL* mutant (□). T_0_ is the end of exponential phase, and Tn is *n* hours after T_0_. **(A)** Promoter of HD73_5327 with *lacZ* fusion. **(B)** Promoter of HD73_0179 with *lacZ* fusion. **(C)** Promoter of HD73_2025 with *lacZ* fusion. **(D)** Promoter of HD73_4161 with *lacZ* fusion. **(E)** Promoter of HD73_1649 with *lacZ* fusion. **(F)** Promoter of HD73_4943 with *lacZ* fusion.

**Figure 2:**
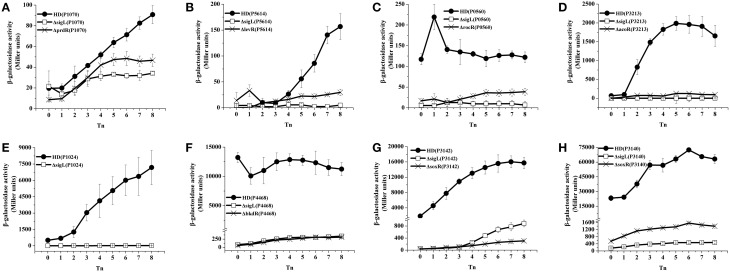
**Identification of EBP target genes**. β-galactosidase activity assay of the −12/−24 promoters in wild-type strain HD73 (•), the *sigL* mutant (□), and the EBP mutants (×). T_0_ is the end of exponential phase, and Tn is n hours after T_0_. **(A)** Promoter of HD73_1070 with *lacZ* fusion. **(B)** Promoter of HD73_5614 with *lacZ* fusion. **(C)** Promoter of HD73_0560 with *lacZ* fusion. **(D)** Promoter of HD73_3213 with *lacZ* fusion. **(E)** Promoter of HD73_1024 with *lacZ* fusion. **(F)** Promoter of HD73_4468 with *lacZ* fusion. **(G)** Promoter of HD73_3142 with *lacZ* fusion. **(H)** Promoter of HD73_3140 with *lacZ* fusion.

To validate the results of the transcriptome and −12/−24 region analyses, 21 promoter fusions with the *lacZ* gene were constructed and analyzed by evaluating β-galactosidase activity. The activation of 16 promoters was abolished and decreased in the Δ*sigL* mutants containing the *gabT* (Zhu et al., 2010) and *kamA* (Zhang et al., 2014) promoters, these promoters are regulated by σ^54^ (Figures 1A–F, 2A–H). Three of these (HD73_3213, HD73_4468, and HD73_5614) were regulated by sequences that were highly similar to *B. subtilis* SigL promoter elements (Debarbouille et al., 1991a, 1999; Ali et al., 2001). One promoter (P2953) containing the highly conserved −12/−24 region was expressed neither in HD73 nor in the Δ*sigL* mutant. The activation of two of the promoters (P2699 and P4960) was unaffected and two (P0035 and P1772) showed increased expression in the Δ*sigL* mutant, suggesting that they are not directly controlled by σ^54^ (data not shown). Finally, the σ^54^ regulon of the Bt HD73 strain included five genes and 11 operons containing a total of 47 genes (Table 2, the annotation of these genes is described in Table 3); of these, 12 had highly conserved −12/−24 promoter sequences and four had minimally conserved sequence. The 16 promoters sequences were aligned and the conserved sequence WYGGHDYRNHDNNWGCD was identified for σ^54^-regulated genes.

**Table 2:** **Genes or operons controlled by σ^54^ in Bt**.

**Genes or operons**	**Functions**	**Promoter −12/−24 Sequence**	Position^*^	**EBPs**
HD73_0366-0369	γ-aminobutyric acid pathway	TT**GG**CATACATTTT**GC**A	−55	GabR
HD73_0560-0562	Arginine degradative pathway	TT**GG**TACGTATTTT**GC**A	−34	RocR
HD73_1024-1025	Arginine and proline metabolism	TT**GG**CATGATATTT**GC**A	−37	unknown
HD73_1070	Glutamine amidotransferase, class I	TT**GG**CACGATATTT**GC**T	−152	PrdR
HD73_2540-2541	L-lysine metabolic pathway	TT**GG**CATAACTATT**GC**T	−38	KamR
HD73_3140-3138	Sarcosine metabolic pathway	TT**GG**CATGATTTTT**GC**A	−41	SoxR
HD73_3142-3147	Sarcosine metabolic pathway	TT**GG**CACGTCAATT**GC**A	−41	SoxR
HD73_3213-3217	Acetoin degradative pathway	TT**GG**CACGGTACTT**GC**A	−37	AcoR
HD73_4161	Proline metabolism	TT**GG**CACGCTATTT**GC**T	−32	Unknown
HD73_4468-4462	Isoleucine and valine degradation pathway	TT**GG**CACGGTATTT**GC**T	−44	BkdR
HD73_5327	Ubiquinone and terpenoid-quinone biosynthesis	TT**GG**CATATATGCT**GC**A	−613	Unknown
HD73_5614-5613	PTS system	TT**GG**CACGCTAATT**GC**A	−387	LevR
HD73_0179-0178	Arginine and proline metabolism	TT**GG**TATGACAAAA**GC**A	−289	Unknown
HD73_1649	Lysine biosynthesis	TT**GG**AGATGTTGAT**GC**G	−169	Unknown
HD73_2025-2030	Valine, leucine, and isoleucine metabolism	TC**GG**AGCATCGCTT**GC**G	−590	Unknown
HD73_4943	Acetate-CoA ligase	AT**GG**CTTAGAAAGA**GC**G	−166	Unknown

* *Distance between the −12 region of the promoter relative to the initiation codon*.

**Table 3:** **Genes controlled by σ^54^ in *B. thuringiensis* HD73**.

**Genes ID**	**Annotation**	**Fold-change**
HD73_0366	4-aminobutyrate aminotransferase	9.165
HD73_0367	Sensory box sigma-54 dependent DNA-binding response regulator	2.860
HD73_0368	Succinic semialdehyde dehydrogenase	2.410
HD73_0369	Quaternary ammonium compound-resistance protein	2.377
HD73_0560	Biotin carboxyl carrier protein	-
HD73_0561	Hypothetical protein	-
HD73_0562	Amino-acid permease rocC	-
HD73_1024	Proline racemase	62.621
HD73_1025	Hypothetical protein	3.687
HD73_1070	Glutamine amidotransferase, class I	5.429
HD73_2540	L-lysine 2,3-aminomutase	2.685
HD73_2541	Cytoplasmic protein	2.296
HD73_3140	Hypothetical protein	73.623
HD73_3139	Hypothetical protein	80.805
HD73_3138	Sarcosine oxidase alpha subunit	18.422
HD73_3142	Sarcosine oxidase, beta subunit	35.033
HD73_3143	Proline racemase	35.278
HD73_3144	Hypothetical protein	31.999
HD73_3145	Dihydrodipicolinate synthase	34.853
HD73_3146	Aldehyde dehydrogenase	28.844
HD73_3147	Amino acid carrier protein	37.362
HD73_3213	Acetoin:2,6-dichlorophenolindophenol oxidoreductase subunit alpha	25.424
HD73_3214	TPP-dependent acetoin dehydrogenase E1 alpha-subunit	19.979
HD73_3215	Acetoin:2,6-dichlorophenolindophenol oxidoreductase subunit beta	9.610
HD73_3216	Dihydrolipoyllysine-residue acetyltransferase component of acetoin cleaving system	9.326
HD73_3217	Dihydrolipoyl dehydrogenase	4.337
HD73_4161	Proline dipeptidase	103.525
HD73_4468	Phosphate butyryltransferase	6.110
HD73_4467	Leucine dehydrogenase	-
HD73_4466	Branched-chain-fatty-acid kinase	2.728
HD73_4465	Dihydrolipoamide dehydrogenase	-
HD73_4464	BfmBAa	-
HD73_4463	2-oxoisovalerate dehydrogenase subunit beta	-
HD73_4462	Lipoamide acyltransferase component of branched-chain alpha-keto acid dehydrogenase complex	-
HD73_5327	NADPH dehydrogenase, quinone	-
HD73_5613	PTS system cellobiose-specific IIC component	0.460
HD73_5614	PTS system cellobiose-specific IIC component	0.460
HD73_0179	Pyrroline-5-carboxylate reductase	2.125
HD73_0178	YtbE (Aldo/keto reductase YtbE)	2.024
HD73_1649	Diaminopimelate decarboxylase	2.270
HD73_2025	Branched-chain amino acid aminotransferase	3.868
HD73_2026	Hypothetical protein	4.825
HD73_2027	acetolactate synthase 1 regulatory subunit	4.073
HD73_2028	Ketol-acid reductoisomerase	4.921
HD73_2029	Dihydroxy-acid dehydratase	3.666
HD73_2030	IlvA	3.120
HD73_4943	Acetate-CoA ligase	2.250

*-, No detection in DNA microarray*.

### Prokaryotic EBPS encoded by the Bt genome

**Figure 3:**
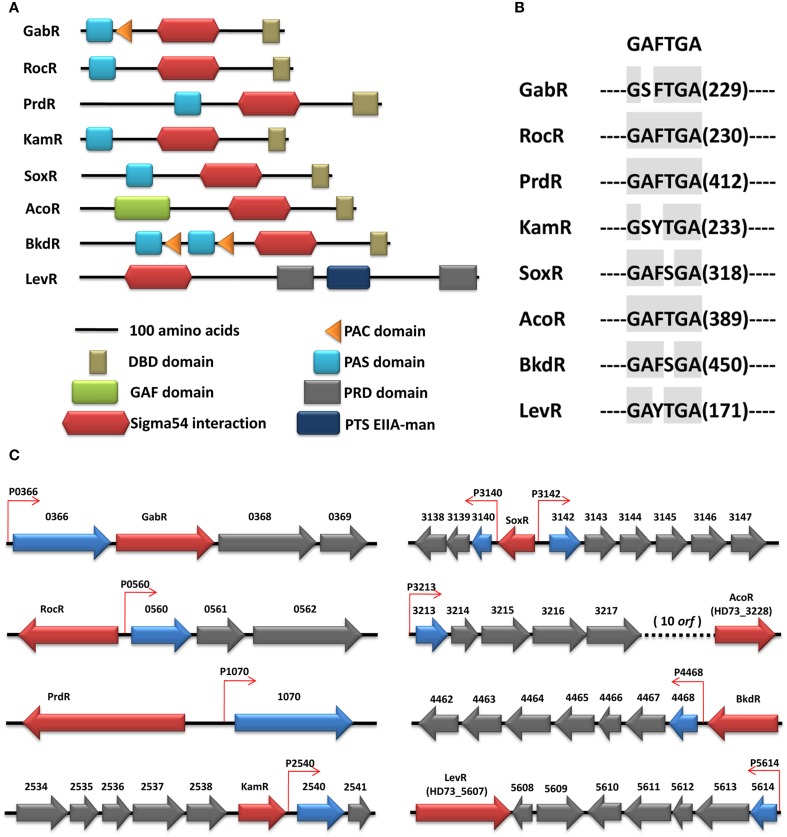
**(A)** Domain structure of the EBPs in the Bt HD73 genome. The type of domains is indicated. All proteins are drawn at the scale indicated. **(B**) Conserved domain of the EBPs. The number indicates the distance between the conserved domain relative to the first amino acid. **(C)** Chromosomal context of EBPs in the Bt HD73 *g*enome. The chromosomal context of those EBPs which have an associated σ^54^ promoter is shown here. Genes encoding EBPs are colored in red, whereas the genes or the first gene of the operons controlled by σ^54^ are represented in blue. The −12/−24 promoters are represented as small arrows and colored in red.

The activation of bEBPs is required for σ^54^-dependent transcription, and the identification of these proteins is therefore important in order to obtain a complete description of the σ^54^ regulon. Using hidden Markov models, eight bEBPs were identified in the HD73 genome with σ^54^ activator domains (Figure 3A). Four different N-terminal regulatory domain arrangements were identified in these eight proteins: those with a Per, ARNT, and Sim (PAS) domain (Ponting and Aravind, 1997), either alone or in combination with an aspartokinase, chorismate mutase, and TyrA (ACT or PAC) domain (Bush and Dixon, 2012); a cyclic GMP [cGMP]-specific and -stimulated phosphodiesterases, *Anabaena* adenylate cyclases, and *E. coli* FhlA (GAF) domain (Bush and Dixon, 2012); PTS regulatory domains (PRDs); and a PTS system fructose IIA component (PTS EIIA-man). PAS domains were identified in six σ^54^-dependent activators: HD73_0367 (GabR), HD73_0559 (RocR), HD73_1069 (PrdR), HD73_2539 (KamR), HD73_3141 (SoxR), and HD73_4469 (BkdR) (Figure 3A). GabR and BkdR also contained PAC domains; interestingly, the latter had two PAS/PAC domains, possibly for sensing different signals. Only one σ^54^-dependent activator, HD73_3228 (AcoR), harbored a GAF domain. Two alternative regulatory domains—a PRD domain and PTS EIIA-man—were present in HD73_5607 (LevR). Sequence alignments of bEBP AAA+ domains revealed a highly conserved GAFTGA motif (Zhang et al., 2009), in all eight putative bEBPs of Bt HD73 (Figure 3B), reflecting its importance for σ^54^- dependent transcription.

An important feature of σ^54^-dependent activators is their tendency to map close to their target promoters (Studholme, 2002). That was indeed the case for the majority of the Bt HD73 regulators, which had three typical organizations (Figure 3C). The most frequently observed was one in which the target promoter was located in the same direction as the activator (GabR, KamR, AcoR, and BkdR); alternatively, σ^54^-dependent promoters could be located immediately up- or downstream of the activator in the opposite orientation (e.g., RocR, PrdR, and LevR); in a third arrangement, the activator was located between two target promoters (e.g., SoxR).

### Identification of EBP target genes

Several bEBP target genes were identified in the present work. We previously characterized the expression of the GABA pathway gene cluster in which *gabT* (HD73_0366) is controlled by σ^54^ and activated by GabR (Zhu et al., 2010), which specifically bound to a repeat region 58 bp upstream of the start codon (Peng et al., 2014). HD73_2540 (*kamA*, encoding l-lysine 2, 3-aminomutase) and its six upstream genes (HD73_2539–2534) form two operons. The activation of the *kamA* promoter—which is a typical −12/−24 sequence—was abolished in the Δ*sigL* mutant from T_0_ to T_4_ and was reduced from T_4_ to T_7_ as compared to the HD73 strain. The promoter was activated by KamR prior to T_7_ and controlled by σ^K^ and GerE after T_10_, the late stage of sporulation (Zhang et al., 2014).

The other eight typical −12/−24 promoters (P0560, P1024, P1070, P3140, P3142, P3213, P4468, and P5614) were analyzed to determine whether they are activated by their neighboring σ^54^-dependent activator. Mutants of six bEBPs (*rocR*, *prdR*, *soxR, acoR*, *bkdR*, and *levR*) were generated by homologous recombination and the expression of putative −12/−24 promoters was assessed with *lacZ* fusion constructs in each mutant background. The β-galactosidase assays showed that the activation of P0560, P1070, P3140, P3142, P3213, P4468, and P5614 was markedly decreased in the corresponding EBP mutants (*rocR*, *prdR*, *soxR*, *acoR*, *bkdR*, and *levR*, respectively), and these seven promoters were also controlled by σ^54^ (Figures 2A–D,F–H). Thus, RocR, PrdR, SoxR, AcoR, BkdR, and LevR are the EBPs of HD73_0560 (biotin carboxyl carrier protein), HD73_1070 (glutamine amidotransferase), HD73_3140 (hypothetical protein), HD73_3142 (sarcosine oxidase, β subunit), HD73_3213 (acetoin, 2, 6-dichlorophenolindophenol oxidoreductase subunit alpha), HD73_4468 (phosphate butyryltransferase), and HD73_5614 (PTS system cellobiose-specific IIC component), respectively. As an exception, the activation of P1024—which is also a typical −12/−24 promoter—was abolished in the Δ*sigL* but was unaltered in the *prdR* mutant, suggesting that PrdR is not the EBP of HD73_1024 (proline racemase).

## Discussion

In this work, computational predictions combined with a functional analysis were used to investigate the Bt HD73 σ^54^ regulon. This regulon comprises five genes and 11 operons for a total of 47 genes, most of which possess typical −12/−24 promoters and are present in other *B. cereus* group strains, with the exception of HD73_1070 (glutamine amidotransferase) which is present in only a few strains such as FRI-35 (Additional file 3). These findings suggest that the σ^54^ regulon is conserved in the *B. cereus* group and provide insights into evolutionary relationships among its members, as well as mechanisms of metabolic regulation that contribute to host range. Most genes in the σ^54^ regulon are related to nitrogen- and carbon-metabolism pathways. Significantly, four operons (*aco*, *bkd*, *lev*, and *roc*) are involved in the acetoin catabolic pathway, isoleucine and valine utilization, generating sugar synthesis metabolites, and arginine catabolism, which is similar to *B. subtilis* in terms of putative function. These operons are controlled by σ^54^ and are regulated by the AcoR, BkdR, LevR, and RocR proteins both in Bt and *B. subtilis* (Debarbouille et al., 1991a, 1999; Ali et al., 2001, 2003). Interestingly, five other σ^54^-dependent genes or gene clusters, which are regulated by four EBPs (GabR, PrdR, KamR, and SoxR), have a unique organization in the *B. cereus* group. These σ^54^-dependent genes are involved in the GABA pathway (Zhu et al., 2010); glutamine, l-lysine (Zhang et al., 2014), and sarcosine metabolism; and are controlled by other σ^70^–type Sigma factors in some bacteria (Nishiya et al., 1998; Schneider et al., 2002; Errington, 2003; Belitsky and Sonenshein, 2004; Steil et al., 2005). The remaining seven σ^54^-dependent operons, which were not recognized by the corresponding EBPs, are involved in arginine and proline metabolism, lysine biosynthesis, branched-chain amino acids biosynthesis, ubiquinone- and other terpenoid-quinone biosynthesis reactions, and catabolism by acetate-CoA ligase. Our data reveals for the first time the seven operons mentioned above are controlled by σ^54^ in the *B. cereus* group.

σ^54^-mediated control of transcription is not only associated with nitrogen and carbon metabolism, but with a wider range of cellular processes and physiology in the bacteria. It was shown that its role also encompasses the regulation of flagellar biosynthesis in *E. coli* and *Geobacter sulfurreducens* (Leang et al., 2009; Zhao et al., 2010); osmotolerance in *Listeria* (Okada et al., 2008); and motility, biofilm formation, luminescence, and colonization in *Vibrio fischeri* (Wolfe et al., 2004; Visick, 2009). We were reported that transcription of the lysine-2, 3-aminomutase gene in the *kam* locus of Bt is controlled both by σ^54^ and the RNA polymerase sigma factor σ^K^, which is associated with late-stage sporulation (Zhang et al., 2014). The mutation of *kamR* slightly decreased the sporulation rate, suggesting that the metabolic pathways regulated by KamR may be involve in sporulation (Zhang et al., 2014). Herein, data from the DNA microarray analysis revealed 255 genes that were downregulated and 121 that were upregulated in the Δ*sigL* mutant relative to the wild-type strain. Among the downregulated genes, 17 genes were associated with sporulation (Additional file 1) but lacked typical −12/−24 promoters, suggesting that σ^54^ may play a role in sporulation by controlling metabolic pathways in the *B. cereus* group and may indirectly regulate some sporulation related genes. These findings should open very interesting avenues of investigation in future studies.

The transcription of many σ^54^-controlled genes involved in physiological processes is induced or repressed by various environmental signals, which are mediated by the N-terminal domains of bEBPs and thereby regulate the activity of the central AAA^+^ domain (Bush and Dixon, 2012). For example, the regulatory domain of FhlA binds formate to activate transcription of the formate hydrogen lyase system in *E. coli* (Hopper and Bock, 1995). The acetoin catabolic pathway, which is activated by AcoR, is induced by acetoin in *B. subtilis* (Ali et al., 2001). The σ^54^-dependent *bkd* and *roc* operons were most strongly and specifically induced after ammonium starvation (Tam Le et al., 2007). Our previous studies also showed that the specific signaling factors GABA and succinic semialdehyde activated expression of *gabT*, which encodes the GABA transaminase of the GABA pathway. Its inducible activation is controlled by σ^54^ and regulated by GabR, and the PAS domain in GabR represses its enhancer transcriptional activity of the *gabT* promoter (Peng et al., 2014). However, in this study, the DNA microarray data was generated from cells grown in SSM medium without specific inducers of σ^54^-regulated promoters, containing only a nutrient broth with a low concentration of metal salts. Relative to the wild-type strain, the differentially expressed genes in the Δ*sigL* mutant reflect the basal gene expression levels. It will be interesting to compare the effects of *sigL* mutations and EBP mutations under inducing conditions in future work, which may reveal that additional σ^54^-dependent genes participate in physiological functions.

## Author contributions

QP and FS designed the research. QP performed the experimental work and drafted the manuscript. QP constructed the promoter with *lacZ* fusions strain and carried out β-galactosidase assay. GW constructed the EBP mutants. GL participated in DNA microarray analysis. FS and JZ critically revised the manuscript for intellectual content. All authors read and approved the final version of the manuscript.

### Conflict of interest statement

The authors declare that the research was conducted in the absence of any commercial or financial relationships that could be construed as a potential conflict of interest.
