# Supplementary material for: Identification of metabolism pathways directly regulated by sigma54 factor in Bacillus thuringiensis
Source: Front Microbiol. 2015 May 12;6:407. doi: 10.3389/fmicb.2015.00407 (PMC4428206; doi:10.3389/fmicb.2015.00407)
Supplement: Supplementary file 1 [file Table1.PDF]

Additional file 1. Functional classification of down- and upregulated genes expressed in the *ΔsigL* mutant

**Downregulated genes**			
**Functions**	**Genes ID**	**Annotations**	**Fold-change**
**Amino acid metabolism**	HD73_0178	YtbE (Aldo/keto reductase YtbE)	2.024
	HD73_0179	Pyrroline-5-carboxylate reductase	2.125
	HD73_0218	4-hydroxyphenylpyruvate dioxygenase	2.161
	HD73_0221	Amino acid transporter	4.192
	HD73_0352	1-pyrroline-5-carboxylate dehydrogenase	2.491
	HD73_0366	4-aminobutyrate aminotransferase	9.165
	HD73_0367	Sensory box sigma-54 dependent DNA-binding response regulator	2.860
	HD73_0368	succinic semialdehyde dehydrogenase	2.410
	HD73_0369	quaternary ammonium compound-resistance protein	2.377
	HD73_0486	amino acid ABC transporter, ATP-binding protein	3.247
	HD73_0673	Bacillolysin	2.423
	HD73_0952	Amino acid transporter	6.536
	HD73_0962	Transporter, EamA	16.989
	HD73_0964	hypothetical protein	4.634
	HD73_1024	Proline racemase	62.621
	HD73_1025	hypothetical protein	3.687
	HD73_1070	Glutamine amidotransferase, class I	5.429
	HD73_1465	hypothetical protein	29.743
	HD73_1466	anthranilate phosphoribosyltransferase	9.128
	HD73_1467	Indole-3-glycerol phosphate synthase	7.691
	HD73_1468	N-(5'-phosphoribosyl)anthranilate isomerase	7.820
	HD73_1469	Tryptophan synthase beta chain	4.407
	HD73_1470	Tryptophan synthase alpha chain	4.528
	HD73_1488	Dihydrolipoyllysine-residue succinyltransferase component of 2-oxoglutarate dehydrogenase complex	2.225
	HD73_1626	Branched-chain amino acid aminotransferase	2.814
	HD73_1627	hypothetical protein	7.128
	HD73_1628	acetolactate synthase 3 regulatory subunit	10.439
	HD73_1629	Ketol-acid reductoisomerase	12.205
	HD73_1630	2-isopropylmalate synthase	13.443
	HD73_1631	3-isopropylmalate dehydrogenase	14.504
	HD73_1632	3-isopropylmalate dehydratase large subunit	15.158
	HD73_1633	isopropylmalate isomerase small subunit	13.217
	HD73_1639	1-(5-phosphoribosyl)-5-[(5-phosphoribosylamino)methylideneamino] imidazole-4-carboxamide isomerase	2.243
	HD73_1640	imidazoleglycerol phosphate synthase cyclase subunit	2.083
	HD73_1641	phosphoribosyl-AMP cyclohydrolase	2.017
	HD73_1649	Diaminopimelate decarboxylase	2.270
	HD73_2025	branched-chain amino acid aminotransferase	3.868
	HD73_2026	hypothetical protein	4.825

Downregulated genes			
**Functions**	**Genes ID**	**Annotations**	**Fold-change**
**Amino acid metabolism**	HD73_2027	acetolactate synthase 1 regulatory subunit	4.073
	HD73_2028	Ketol-acid reductoisomerase	4.921
	HD73_2029	Dihydroxy-acid dehydratase	3.666
	HD73_2030	IlvA	3.120
	HD73_2092	Branched-chain amino acid ABC transporter, substrate-binding protein	6.432
	HD73_2093	Branched-chain amino acid ABC transporter, ATP-binding protein	8.995
	HD73_2094	hypothetical protein	5.797
	HD73_2095	branched chain amino acid ABC transporter permease	2.395
	HD73_2096	Branched chain amino acid ABC transporter, permease	2.064
	HD73_2450	Bacillolysin	4.821
	HD73_2540	L-lysine 2,3-aminomutase	2.685
	HD73_2541	cytoplasmic protein	2.296
	HD73_3138	Sarcosine oxidase alpha subunit	18.422
	HD73_3139	hypothetical protein	80.805
	HD73_3140	hypothetical protein	73.623
	HD73_3142	Sarcosine oxidase beta subunit	35.033
	HD73_3143	proline racemase	35.278
	HD73_3144	hypothetical protein	31.999
	HD73_3145	Dihydrodipicolinate synthase	34.853
	HD73_3146	Aldehyde dehydrogenase	28.844
	HD73_3147	amino acid carrier protein	37.362
	HD73_3917	urease subunit alpha	2.245
	HD73_4008	GABA permease	5.023
	HD73_4161	Proline dipeptidase	103.525
	HD73_4925	argininosuccinate synthase	2.750
	HD73_5406	Neutral protease B	3.146
	HD73_5817	homoserine dehydrogenase	4.759
	HD73_5818	homoserine O-succinyltransferase	2.094
**Carbohydrate metabolism**	HD73_0505	Hydroxyethylthiazole kinase	2.725
	HD73_1525	hypothetical protein	2.083
	HD73_1545	acetoacetyl-CoA reductase	2.541
	HD73_2460	Phosphoglycolate phosphatase	2.004
	HD73_3213	Acetoin:2,6-dichlorophenolindophenol oxidoreductase subunit alpha	25.424
	HD73_3214	TPP-dependent acetoin dehydrogenase E1 alpha-subunit	19.979
	HD73_3215	Acetoin:2,6-dichlorophenolindophenol oxidoreductase subunit beta	9.610
	HD73_3216	Dihydrolipoyllysine-residue acetyltransferase component of acetoin cleaving system	9.326
	HD73_3217	Dihydrolipoyl dehydrogenase	4.337

Downregulated genes			
**Functions**	**Genes ID**	**Annotations**	**Fold-change**
**Carbohydrate metabolism**	HD73_3759	Phosphatidylinositol-specific phospholipase	2.757
	HD73_3826	Aldehyde dehydrogenase	2.810
	HD73_4009	hypothetical protein	3.506
	HD73_4119	SucC	2.035
	HD73_4223	formamidase	4.374
	HD73_4943	Acetate-CoA ligase	2.250
	HD73_4965	Acetyl-coenzyme A synthetase	3.841
	HD73_5341	putative phosphoglycerate mutase family protein	2.844
**Sporulation**	HD73_0035	AbrB	2.382
	HD73_0061	stage II sporulation protein E	2.020
	HD73_0093	RNA polymerase sigma-H factor	2.307
	HD73_1411	oligopeptide ABC transporter, permease protein	3.244
	HD73_1412	oligopeptide transport system permease oppC	3.182
	HD73_1413	oligopeptide ABC transporter ATP-binding protein	3.765
	HD73_1511	SinR protein	2.403
	HD73_1512	SinI protein	2.772
	HD73_2139	Polysaccharide deacetylase	2.899
	HD73_2203	Oligopeptide ABC transporter, oligopeptide-binding protein	2.747
	HD73_3960	MutT/NUDIX	2.071
	HD73_4180	cell division protein DivIVA	2.047
	HD73_4489	stage III sporulation protein AH	2.975
	HD73_4490	stage III sporulation protein AG	2.662
	HD73_4495	stage III sporulation protein AB	2.553
	HD73_4496	stage III sporulation protein AA	2.305
	HD73_4789	Spore germination protein gerM	2.405
**Transport system**	HD73_0653	Transporter, drug/metabolite exporter	3.678
	HD73_0715	ABC transporter protein	2.151
	HD73_0742	RbsB (Ribose ABC transporter)	2.497
	HD73_0872	Potassium-transporting ATPase A chain	4.391
	HD73_0873	Potassium-transporting ATPase A chain	2.971
	HD73_0875	Potassium-transporting ATPase C chain	3.111
	HD73_0934	hypothetical protein	7.075
	HD73_0935	ABC transporter, permease protein	3.528
	HD73_1507	Signal peptidase I	2.006
	HD73_1661	Peptidase, family M23/M37	2.466
	HD73_1662	proton/glutamate symporter family protein	2.339
	HD73_1843	putative symporter ywcA	2.325
	HD73_2595	Drug resistance transporter, EmrB/QacA	5.281
	HD73_2966	proline/betaine transporter	3.685
	HD73_2967	hypothetical protein	2.066
	HD73_3698	putative transporter	2.342
	HD73_3903	BioY	4.854

Downregulated genes			
**Functions**	**Genes ID**	**Annotations**	**Fold-change**
**Transport system**	HD73_4020	iron ABC transporter permease	2.724
	HD73_4021	Iron-uptake system permease protein feuB	3.378
	HD73_4022	Iron(III) dicitrate-binding protein	2.055
	HD73_4023	hypothetical protein	3.218
	HD73_4298	Maltosaccharide ABC transporter, maltosaccharide-binding protein	2.060
	HD73_4371	Sodium-dependent transporter	8.088
	HD73_4372	hypothetical protein	6.610
	HD73_4675	hypothetical protein	3.476
	HD73_4676	Iron compound ABC transporter, permease protein	3.201
	HD73_5215	bioY family protein	3.660
	HD73_5451	ferric anguibactin transport system permease fatC	2.350
	HD73_5661	Efflux transporter, RND family, MFP subunit	2.445
**Signal transduction**	HD73_0529	Tellurium resistance protein	2.498
	HD73_0530	tellurium resistance protein, TerD	2.265
	HD73_1056	Two-component sensor kinase citS	2.792
	HD73_1057	hypothetical protein	2.266
	HD73_2138	hypothetical protein	3.005
	HD73_2857	hypothetical protein	2.920
	HD73_3564	TIGR00366 family protein	5.007
	HD73_4466	Branched-chain-fatty-acid kinase	2.728
	HD73_4468	phosphate butyryltransferase	6.110
**Other functions**	HD73_0098	RplJ	2.119
	HD73_0109	30S ribosomal protein S10	2.572
	HD73_0111	50S ribosomal protein L4	2.013
	HD73_0113	50S ribosomal protein L2	2.299
	HD73_0114	30S ribosomal protein S19	2.663
	HD73_0115	50S ribosomal protein L22	2.522
	HD73_0116	30S ribosomal protein S3	2.025
	HD73_0131	adenylate kinase	2.012
	HD73_0399	hypothetical protein	2.204
	HD73_0401	hypothetical protein	2.747
	HD73_0458	NADH dehydrogenase	2.069
	HD73_0483	Fatty acid desaturase	2.059
	HD73_0521	hypothetical protein	2.620
	HD73_0634	Ankyrin repeat domain protein	2.115
	HD73_0744	Immune inhibitor A	2.564
	HD73_0818	3D domain protein	2.607
	HD73_0826	Nucleic acid binding OB-fold tRNA/helicase-type	3.074
	HD73_0854	hypothetical protein	2.014
	HD73_0929	3D domain protein	2.739
	HD73_0988	hypothetical protein	2.650

Downregulated genes			
**Functions**	**Genes ID**	**Annotations**	**Fold-change**
**Other functions**	HD73_1008	O-succinylbenzoate-CoA ligase	2.455
	HD73_1016	3-hydroxybutyryl-CoA dehydratase	2.349
	HD73_1090	hypothetical protein	2.972
	HD73_1121	hypothetical protein	2.186
	HD73_1142	hypothetical protein	3.573
	HD73_1143	hypothetical protein	2.607
	HD73_1144	hypothetical protein	3.799
	HD73_1153	hypothetical protein	2.854
	HD73_1220	Antibiotic biosynthesis monooxygenase	2.797
	HD73_1225	hypothetical protein	2.358
	HD73_1301	ATP-dependent helicase/deoxyribonuclease subunit B	2.354
	HD73_1455	Acetyltransferase	2.450
	HD73_1456	hypothetical protein	3.350
	HD73_1474	hypothetical protein	3.515
	HD73_1509	hypothetical protein	3.580
	HD73_1524	SCP-like extracellular	4.343
	HD73_1544	hypothetical protein	2.227
	HD73_1596	hypothetical protein	2.058
	HD73_1698	D-alanyl-D-alanine carboxypeptidase family protein	2.371
	HD73_1752	menaquinol-cytochrome c reductase iron-sulfur subunit	2.033
	HD73_1753	cytochrome b6	2.331
	HD73_1757	hypothetical protein	2.086
	HD73_1772	3-methyl-2-oxobutanoate hydroxymethyltransferase	2.123
	HD73_1797	Ribosomal-protein-alanine acetyltransferase	2.041
	HD73_1845	hypothetical protein	2.006
	HD73_1948	hypothetical protein	3.414
	HD73_2063	Pyn	2.203
	HD73_2083	2-hydroxychromene-2-carboxylate isomerase	2.644
	HD73_2118	putative cell wall peptidase, NlpC/P60 family	4.798
	HD73_2211	alkaline phosphatase	2.309
	HD73_2224	hypothetical protein	7.977
	HD73_2233	Acetyltransferase, GNAT	2.033
	HD73_2321	hypothetical protein	2.168
	HD73_2396	hypothetical protein	2.237
	HD73_2454	aspartate-tRNA ligase	2.292
	HD73_2589	2,3-dihydro-2,3-dihydroxybenzoate dehydrogenase	2.568
	HD73_2591	2,3-dihydroxybenzoate-AMP ligase	2.988
	HD73_2593	Dimodular nonribosomal peptide synthetase	5.658
	HD73_2598	DNA-binding protein HU	2.545
	HD73_2756	hypothetical protein	2.895
	HD73_2832	General stress protein 17M	2.814
	HD73_2833	hypothetical protein	2.526

Downregulated genes			
**Functions**	**Genes ID**	**Annotations**	**Fold-change**
**Other functions**	HD73_2852	hypothetical protein	2.505
	HD73_2854	hemolysin BL binding component B	2.218
	HD73_2869	hypothetical protein	2.825
	HD73_2907	hypothetical protein	2.147
	HD73_2910	aminoglycoside phosphotransferase	3.766
	HD73_2911	phosphatase family protein	2.760
	HD73_2912	Uridine kinase	2.601
	HD73_2944	hypothetical protein	2.518
	HD73_2953	hypothetical protein	2.485
	HD73_2968	hypothetical protein	2.015
	HD73_2969	hypothetical protein	2.248
	HD73_2975	hypothetical protein	3.225
	HD73_2977	Acetyltransferase, GNAT	2.786
	HD73_2978	hypothetical protein	2.039
	HD73_2987	Fatty acid desaturase (Membrane bound delta 5 acyl lipid desaturase)	6.688
	HD73_3118	Xaa-Pro dipeptidyl-peptidase	3.581
	HD73_3166	ATP-dependent DNA helicase RecQ	2.231
	HD73_3167	hypothetical protein	2.037
	HD73_3219	hypothetical protein	3.489
	HD73_3273	Lipase	4.285
	HD73_3398	Lipase	6.999
	HD73_3590	Short-chain dehydrogenase/reductase	2.054
	HD73_3609	hypothetical protein	2.633
	HD73_3778	TPR domain protein	5.934
	HD73_3779	hypothetical protein	4.210
	HD73_3783	hypothetical protein	2.076
	HD73_3811	hypothetical protein	3.180
	HD73_3901	acetyl-CoA C-acetyltransferase	2.228
	HD73_3902	hypothetical protein	10.364
	HD73_3909	Acetyltransferase	4.031
	HD73_3943	hypothetical protein	2.765
	HD73_3961	hypothetical protein	4.507
	HD73_4000	3D domain protein	2.168
	HD73_4159	Calcium-translocating P-type ATPase PMCA-type	2.137
	HD73_4302	hypothetical protein	2.351
	HD73_4416	Biotin synthase	4.942
	HD73_4418	hypothetical protein	9.899
	HD73_4419	8-amino-7-oxononanoate synthase	14.241
	HD73_4420	Dethiobiotin synthetase	19.846
	HD73_4421	Adenosylmethionine--8-amino-7-oxononanoate aminotransferase	22.542

Downregulated genes			
**Functions**	**Genes ID**	**Annotations**	**Fold-change**
**Other functions**	HD73_4470	hypothetical protein	2.784
	HD73_4824	DinB	2.895
	HD73_4841	Cell surface protein	3.388
	HD73_4842	Cell surface protein	4.111
	HD73_4843	Iron transport associated protein	3.877
	HD73_4901	hypothetical protein	2.001
	HD73_4960	Tyrosyl-tRNA synthetase	2.120
	HD73_5294	Prophage helix-turn-helix protein	3.979
	HD73_5638	Enterotoxin	2.553
	HD73_5729	nitrilotriacetate monooxygenase component B	2.796
	HD73_5768	Aminopeptidase	6.502
	HD73_5771	hypothetical protein	3.018
	HD73_5801	hypothetical protein	3.243
	HD73_5836	hypothetical protein	2.083
	HD73_6016	Transposase for insertion sequence element IS231B	2.428
**Upregulated genes**			
**Functions**	**Genes ID**	**Annotations**	**Fold-change**
**Amino acid metabolism**	HD73_0088	serine O-acetyltransferase	0.402
	HD73_1416	hypothetical protein	0.476
	HD73_4173	Aspartate carbamoyltransferase	0.351
	HD73_4174	Uracil permease	0.428
	HD73_4952	Methionine gamma-lyase	0.335
**Carbohydrate metabolism**	HD73_0514	hypothetical protein	0.450
	HD73_0924	PTS system diacetylchitobiose-specific IIB component	0.279
	HD73_2573	2-methylcitrate dehydratase	0.305
	HD73_3006	putative aldolase lsrF	0.474
	HD73_3474	acetyl-CoA carboxylase biotin carboxyl carrier protein subunit	0.455
	HD73_3475	putative acetyl-CoA carboxylase biotin carboxylase	0.427
	HD73_3476	acyl-CoA dehydrogenase	0.419
	HD73_4889	Citrate synthase 2	0.375
	HD73_5011	Pullulanase	0.367
	HD73_5083	Phosphoglycolate phosphatase	0.358
	HD73_5613	PTS system cellobiose-specific IIC component	0.460
	HD73_5614	LicB	0.360
	HD73_5615	PTS system lichenan oligosaccharide-specific IIA component	0.474
	HD73_5616	PTS system cellobiose-specific IIC component	0.398
	HD73_5670	UDP-glucose 6-dehydrogenase	0.439
**Sporulation**	HD73_1790	spore coat protein D	0.358
	HD73_2563	Spore germination protein PF	0.432
**Transport system**	HD73_0177	Oligopeptide transporter periplasmic-binding protein	0.418
	HD73_0354	ABC transporter ATP-binding protein	0.319
	HD73_0355	ABC transporter permease	0.250

Upregulated genes			
**Functions**	**Genes ID**	**Annotations**	**Fold-change**
**Transport system**	HD73_0454	NhaC family sodium:proton (Na+:H+) antiporter	0.355
	HD73_1217	putative transporter	0.431
	HD73_3010	ABC transporter	0.350
	HD73_3011	hypothetical protein	0.435
	HD73_3012	hypothetical protein	0.390
	HD73_3013	hypothetical protein	0.320
	HD73_3561	Quinolone resistance protein major facilitator family transporter	0.440
	HD73_3859	Extracellular solute-binding protein family 5	0.499
**Signal transduction**	HD73_0460	5-methylthioribose kinase	0.492
	HD73_0493	Crp family transcriptional regulator	0.310
	HD73_0704	Sensory box/GGDEF	0.431
	HD73_1239	hypothetical protein	0.293
	HD73_1240	Transcriptional regulator LacI	0.319
	HD73_1718	adaptor protein	0.460
	HD73_4267	CBS domain-containing protein	0.202
	HD73_4818	Transcriptional regulator TetR	0.436
	HD73_4919	Universal stress protein	0.384
	HD73_5408	Transcriptional regulator (MarR family)	0.482
	HD73_5464	hypothetical protein	0.181
	HD73_5465	Murein hydrolase exporter LrgA	0.113
	HD73_5474	RNA polymerase sigma-54 factor	0.398
	HD73_5530	rha family phage regulatory protein	0.440
	HD73_5853	hypothetical protein	0.339
	HD73_5854	Murein hydrolase exporter	0.269
**Other functions**	HD73_0251	cro repressor	0.260
	HD73_0256	hypothetical protein	0.374
	HD73_0284	Phage portal protein HK97	0.348
	HD73_0288	hypothetical protein	0.494
	HD73_0668	cadmium efflux system accessory protein	0.162
	HD73_0711	Spore germination protein KA	0.483
	HD73_0753	hypothetical protein	0.452
	HD73_0754	hypothetical protein	0.269
	HD73_0838	cytochrome aa3 quinol oxidase polypeptide II	0.449
	HD73_0973	hypothetical protein	0.458
	HD73_1088	hypothetical protein	0.442
	HD73_1179	hypothetical protein	0.344
	HD73_1180	hypothetical protein	0.272
	HD73_1215	hypothetical protein	0.408
	HD73_1216	hypothetical protein	0.413
	HD73_1241	hypothetical protein	0.448
	HD73_1244	hypothetical protein	0.448
	HD73_1280	CAAX amino terminal protease	0.439

Upregulated genes			
**Functions**	**Genes ID**	**Annotations**	**Fold-change**
**Other functions**	HD73_1420	Negative regulator of genetic competence mecA	0.426
	HD73_1499	hypothetical protein	0.439
	HD73_1532	hypothetical protein	0.268
	HD73_1673	Methyltransferase, UbiE/COQ5	0.490
	HD73_1721	hypothetical protein	0.469
	HD73_1788	hypothetical protein	0.157
	HD73_1885	cytoplasmic protein	0.316
	HD73_1886	hypothetical protein	0.217
	HD73_1935	hypothetical protein	0.369
	HD73_1981	Uridylate kinase	0.444
	HD73_2134	hypothetical protein	0.435
	HD73_2187	hypothetical protein	0.301
	HD73_2240	hypothetical protein	0.444
	HD73_2313	putative Membrane Spanning Protein	0.306
	HD73_2402	hypothetical protein	0.496
	HD73_2453	hypothetical protein	0.479
	HD73_2552	hypothetical protein	0.387
	HD73_2553	hypothetical protein	0.372
	HD73_2555	hypothetical protein	0.416
	HD73_2564	hypothetical protein	0.454
	HD73_2600	DinB	0.271
	HD73_2679	glycerol-3-phosphate acyltransferase 1	0.450
	HD73_2759	hypothetical protein	0.477
	HD73_2870	hypothetical protein	0.216
	HD73_3032	hypothetical protein	0.467
	HD73_3089	hypothetical protein	0.426
	HD73_3102	hypothetical protein	0.421
	HD73_3222	hypothetical protein	0.498
	HD73_3351	Glutamate-rich protein grpB	0.383
	HD73_3654	hypothetical protein	0.463
	HD73_3706	Hypothetical protein	0.437
	HD73_3736	hypothetical protein	0.326
	HD73_4205	putative N-acetyltransferase	0.255
	HD73_4312	hypothetical protein	0.306
	HD73_4450	hypothetical protein	0.368
	HD73_4604	hypothetical protein	0.494
	HD73_4819	Long-chain-fatty-acid--CoA ligase	0.476
	HD73_4890	hypothetical protein	0.367
	HD73_5037	hypothetical protein	0.393
	HD73_5250	hypothetical protein	0.483
	HD73_5251	Major tail protein	0.419
	HD73_5254	phage head-tail adaptor	0.447

**Upregulated genes**			
**Functions**	**Genes ID**	**Annotations**	**Fold-change**
**Other functions**	HD73_5255	hypothetical protein	0.329
	HD73_5257	ATP-dependent Clp protease proteolytic subunit ClpP	0.429
	HD73_5261	HNH endonuclease	0.486
	HD73_5281	replication protein O	0.486
	HD73_5286	antirepressor putative	0.478
	HD73_5296	YdcL (Lambda integrase-like, N-terminal,DNA breaking-rejoining enzyme, catalytic core)	0.472
	HD73_5531	hypothetical protein	0.339
	HD73_5658	hypothetical protein	0.470
	HD73_5748	Acyl-CoA dehydrogenase	0.243
	HD73_5750	3-hydroxyacyl-CoA dehydrogenase	0.173
	HD73_5751	Acetyl-CoA acetyltransferase	0.148
	HD73_5752	ferredoxin 4Fe-4S	0.429
