# Supplementary material for: Identification of metabolism pathways directly regulated by sigma54 factor in Bacillus thuringiensis
Source: Front Microbiol. 2015 May 12;6:407. doi: 10.3389/fmicb.2015.00407 (PMC4428206; doi:10.3389/fmicb.2015.00407)
Supplement: Supplementary file 2 [file Table2.PDF]

Additional file 2. Predicted -12/-24 sequence in the complete genome sequence of *B. thuringiensis* HD73

The conserved sequence is BYGGCMYRNNNNYYGCW (Francke et al. 2011)					
**Gene ID**	**Position***	**Sequence**	**Fold-change**	**β-galactosidase activity in *ΔsigL***	**Product**
HD73_0366	-55	TT**GG**CATACATTTT**GC**A	9.165	abolished	4-aminobutyrate aminotransferase
HD73_0560	-34	TT**GG**TACGTATTTT**GC**A	—	abolished	Biotin carboxyl carrier protein
HD73_1024	-37	TT**GG**CATGATATTT**GC**A	62.621	abolished	Proline racemase
HD73_1070	-152	TT**GG**CACGATATTT**GC**T	5.429	decreased	Glutamine amidotransferase, class I
HD73_2540	-38	TT**GG**CATAACTATT**GC**T	2.685	decreased	L-lysine 2,3-aminomutase
HD73_2699	-270	CT**GG**CACGTTTTCC**GC**A	—	unaffected	MarR family transcriptional regulator
HD73_2953	184	TT**GG**CACAGTTTTT**GC**T	2.485	None	hypothetical protein
HD73_3140	-41	TT**GG**CATGATTTTT**GC**A	73.623	decreased	hypothetical protein
HD73_3142	-41	TT**GG**CACGTCAATT**GC**A	35.033	decreased	Sarcosine oxidase, beta subunit
HD73_3213	-37	TT**GG**CACGGTACTT**GC**A	25.424	abolished	Acetoin:2,6-dichlorophenolindophenol oxidoreductase subunit alpha
HD73_4161	-32	TT**GG**CACGCTATTT**GC**T	103.525	abolished	Proline dipeptidase
HD73_4468	-44	TT**GG**CACGGTATTT**GC**T	6.110	abolished	phosphate butyryltransferase
HD73_5327	-613	TT**GG**CATATATGCT**GC**A	—	decreased	NADPH dehydrogenase, quinone
HD73_5614	-387	TT**GG**CACGCTAATT**GC**A	0.460	abolished	PTS system cellobiose-specific IIC component
The minimally conserved sequence NNGGN$_{10}$GCNN					
**Gene ID**	**Position***	**Sequence**	**Fold-change**	**β-galactosidase activity in *ΔsigL***	**Product**
HD73_0035	-344	TT**GG**GCTTTTACGA**GC**A	2.382	increased	AbrB
HD73_0179	-289	TT**GG**TATGACAAAA**GC**A	2.125	decreased	Pyrroline-5-carboxylate reductase
HD73_1649	-169	TT**GG**AGATGTTGAT**GC**G	2.270	decreased	Diaminopimelate decarboxylase
HD73_1772	-98	GA**GG**GATGAATAAT**GC**C	2.123	increased	3-methyl-2-oxobutanoate hydroxymethyltransferase
HD73_2025	-590	TC**GG**AGCATCGCTT**GC**G	3.868	decreased	branched-chain amino acid aminotransferase
HD73_4943	-166	AT**GG**CTTAGAAAGA**GC**G	2.250	abolished	Acetate-CoA ligase
HD73_4960	-150	G**GG**AAAAGACGGT**GC**T	2.120	unaffected	Tyrosyl-tRNA synthetase

*****Distance between the -12 region of the promoter relative to the initiation codon.

—: No detection in DNA microarray.
