# Supplementary material for: Identification of metabolism pathways directly regulated by sigma54 factor in Bacillus thuringiensis
Source: Front Microbiol. 2015 May 12;6:407. doi: 10.3389/fmicb.2015.00407 (PMC4428206; doi:10.3389/fmicb.2015.00407)
Supplement: Supplementary file 3 [file Table3.PDF]

**Additional file 3. Alignment of -12/-24 promoters controlled by $\sigma^{54}$ of Bt HD73 in *Bacillus cereus* group strains**

| Strains | Gene ID | Annotations | -12/-24 sequence | Position* | Identity |
|---|---|---|---|---|---|
| *Bacillus thuringiensis* serovar *kurstaki* HD73 | HD73_0366 | 4-aminobutyrate aminotransferase | TTGGCATACATTTTGCA | -55 | |
| *Bacillus thuringiensis* BMB171 | BMB171_C0300 | 4-aminobutyrate aminotransferase | TTGGCATATATTTTGCA | -43 | 93.82% |
| *Bacillus thuringiensis* 97-27 | BT9727_0293 | 4-aminobutyrate aminotransferase | TTGGCATACATTTTGCA | -43 | 85.83% |
| *Bacillus cereus* ATCC 14579 | BC0355 | 4-aminobutyrate aminotransferase | ATGCAATTGGGGTGCT | -201 | 98.82% |
| *Bacillus cereus* ATCC 10987 | BCE_0354 | 4-aminobutyrate aminotransferase | TTGGCATACATTTTGCA | -1 | 89.24% |
| *Bacillus cereus* E33L | BCZK0297 | 4-aminobutyrate aminotransferase | TTGGCATACATTTTGCA | -43 | 85.63% |
| *Bacillus cereus* FRI-35 | BCK_06285 | 4-aminobutyrate aminotransferase | TTGGCATACATTTTGCA | -43 | 97.10% |
| *Bacillus anthracis* str. Ames | BA_0325 | 4-aminobutyrate aminotransferase | TTGGCATACATTTTGCA | -43 | 85.64% |
| *Bacillus anthracis* A0248 | BAA_0381 | 4-aminobutyrate aminotransferase | TTGGCATACATTTTGCA | 28 | 90.42% |
| *Bacillus anthracis* CDC 684 | BAMEG_0384 | 4-aminobutyrate aminotransferase | TTGGCATACATTTTGCA | 28 | 90.42% |
| *Bacillus mycoides* DSM 2048 | bmyco0001_2780 | 4-aminobutyrate aminotransferase | TTGGCATACATTTTGCA | -31 | 87.15% |
| *Bacillus pseudomycoides* DSM 12442 | bpmyx0001_2610 | 4-aminobutyrate aminotransferase | GTGGCGGTTGTTATGCA | -228 | 78.96% |
| *Bacillus weihenstephanensis* KBAB4 | BcerKBAB4_0305 | 4-aminobutyrate aminotransferase | TTGGCATACATCTTGCA | -43 | 86.04% |

| Strains | Gene ID | Annotations | -12/-24 sequence | Position* | Identity |
|---|---|---|---|---|---|
| *Bacillus thuringiensis* serovar *kurstaki* HD73 | HD73_0560 | Biotin carboxyl carrier protein | TTGGTACGTATTTTGCA | -34 | |
| *Bacillus thuringiensis* BMB171 | BMB171_C0417 | hypothetical protein | TTGGTACGCATTTTGCA | -78 | 92.16% |
| *Bacillus thuringiensis* 97-27 | BT9727_0408 | hypothetical protein | TTGGTATGCTTTTTGCA | -78 | 95.29% |
| *Bacillus cereus* ATCC 14579 | BC0474 | hypothetical protein | TTGGTACGCATTTTGCA | -78 | 92.16% |
| *Bacillus cereus* ATCC 10987 | BCE_0546 | hypothetical protein | TTGGTACGCATTTTGCA | -78 | 94.51% |
| *Bacillus cereus* E33L | BCZK0404 | hypothetical protein | TTGGTACGCATTTTGCA | -78 | 95.29% |
| *Bacillus cereus* FRI-35 | BCK_05665 | hypothetical protein | TTGGTACGCATTTTGCA | -78 | 96.40% |
| *Bacillus anthracis* str. Ames | BA_0490 | hypothetical protein | TTGGTACGCATTTTGCA | -78 | 93.33% |
| *Bacillus anthracis* A0248 | BAA_0552 | hypothetical protein | TTGGTACGCATTTTGCA | -78 | 93.33% |
| *Bacillus anthracis* CDC 684 | BAMEG_4116 | hypothetical protein | TTGGTACGCATTTTGCA | -78 | 93.33% |
| *Bacillus mycoides* DSM 2048 | — | — | — | — | — |
| *Bacillus pseudomycoides* DSM 12442 | — | — | — | — | — |
| *Bacillus weihenstephanensis* KBAB4 | BcerKBAB4_0410 | hypothetical protein | TTGGTACGTATTTTGCA | -106 | 63.66% |

| Strains | Gene ID | Annotations | -12/-24 sequence | Position* | Identity |
|---|---|---|---|---|---|
| *Bacillus thuringiensis* serovar *kurstaki* HD73 | HD73_1024 | Proline racemase | TTGGCATGATATTTGCA | -37 | |
| *Bacillus thuringiensis* BMB171 | BMB171_C0784 | Proline racemase | TTGGATTACCTTCGT | -37 | 76.42% |
| *Bacillus thuringiensis* 97-27 | BT9727_0799 | Proline racemase | TTGGCATGATATTTGCA | -37 | 93.73% |
| *Bacillus cereus* ATCC 14579 | BC0905 | Proline racemase | TTGGCATGATATTTGCA | -37 | 91.44% |
| *Bacillus cereus* ATCC 10987 | BCE_0994 | Proline racemase | TTGGCATGATATTTGCT | -37 | 92.54% |
| *Bacillus cereus* E33L | BCZK0802 | Proline racemase | TTGGCATGATACTTGCA | -37 | 90.65% |
| *Bacillus cereus* FRI-35 | BCK_03710 | proline racemase | TTGGCATGATATTTGCA | -37 | 96.70% |
| *Bacillus anthracis* str. Ames | BA_0901 | Proline racemase | TTGGCATGATATTTGCA | -37 | 91.94% |
| *Bacillus anthracis* A0248 | BAA_1004 | Proline racemase | TTGGCATGATATTTGCA | -37 | 91.94% |
| *Bacillus anthracis* CDC 684 | BAMEG_3661 | Proline racemase | TTGGCATGATATTTGCA | -37 | 91.94% |
| *Bacillus mycoides* DSM 2048 | bmyco0001_7870 | Proline racemase | TTATCCTGCTATTTGTG | -122 | 86.70% |
| *Bacillus pseudomycoides* DSM 12442 | bpmyx0001_7770 | Proline racemase | TTGGCACGGTATTTGCT | -34 | 80.39% |
| *Bacillus weihenstephanensis* KBAB4 | BcerKBAB4_0800 | Proline racemase | TTGGCATGATACTTGCA | -37 | 90.05% |

| Strains | Gene ID | Annotations | -12/-24 sequence | Position* | Identity |
|---|---|---|---|---|---|
| *Bacillus thuringiensis* serovar *kurstaki* HD73 | HD73_1070 | Glutamine amidotransferase, class I | TTGGCACGATATTTGCT | -152 | |
| *Bacillus thuringiensis* BMB171 | — | — | — | — | — |
| *Bacillus thuringiensis* 97-27 | — | — | — | — | — |
| *Bacillus cereus* ATCC 14579 | — | — | — | — | — |
| *Bacillus cereus* ATCC 10987 | — | — | — | — | — |
| *Bacillus cereus* E33L | — | — | — | — | — |
| *Bacillus cereus* FRI-35 | BCK_03535 | peptidase C26 | TTGGCACGATATTTGCT | -152 | 99.60% |
| *Bacillus anthracis* str. Ames | — | — | — | — | — |
| *Bacillus anthracis* A0248 | — | — | — | — | — |
| *Bacillus anthracis* CDC 684 | — | — | — | — | — |
| *Bacillus mycoides* DSM 2048 | — | — | — | — | — |
| *Bacillus pseudomycoides* DSM 12442 | — | — | — | — | — |
| *Bacillus weihenstephanensis* KBAB4 | — | — | — | — | — |

| Strains | Gene ID | Annotations | -12/-24 sequence | Position* | Identity |
|---|---|---|---|---|---|
| *Bacillus thuringiensis* serovar *kurstaki* HD73 | HD73_2540 | L-lysine 2,3-aminomutase | TTGGCATAACTATTGCT | -38 | |
| *Bacillus thuringiensis* BMB171 | BMB171_C2029 | L-lysine 2,3-aminomutase | TTGGCATAACTATTGCT | -38 | 99.44% |
| *Bacillus thuringiensis* 97-27 | BT9727_2083 | L-lysine 2,3-aminomutase | TTGGCATAACTATTGCT | -38 | 94.37% |
| *Bacillus cereus* ATCC 14579 | BC2251 | L-lysine 2,3-aminomutase | TTGGCATAACTATTGCT | -38 | 99.30% |
| *Bacillus cereus* ATCC 10987 | BCE_2334 | L-lysine 2,3-aminomutase | TTGGCATAACTATTGCT | -38 | 94.80% |
| *Bacillus cereus* E33L | BCZK2079 | L-lysine 2,3-aminomutase | TTGGCATAACTATTGCT | -38 | 95.69% |
| *Bacillus cereus* FRI-35 | BCK_23340 | L-lysine 2,3-aminomutase | TTGGCATAACTATTGCT | -38 | 98.70% |
| *Bacillus anthracis* str. Ames | BA_2300 | L-lysine 2,3-aminomutase | TTGGCATAACTATTGCT | -38 | 94.37% |
| *Bacillus anthracis* A0248 | BAA_2364 | L-lysine 2,3-aminomutase | TTGGCATAACTATTGCT | -38 | 94.37% |
| *Bacillus anthracis* CDC 684 | BAMEG_2294 | L-lysine 2,3-aminomutase | TTGGCATAACTATTGCT | -38 | 94.37% |
| *Bacillus mycoides* DSM 2048 | bmyco0001_20160 | L-lysine 2,3-aminomutase | TTGGCATAATTATTGCT | -38 | 90.30% |
| *Bacillus pseudomycoides* DSM 12442 | bpmyx0001_20430 | L-lysine 2,3-aminomutase | TTGGCATAATTATTGCT | -7 | 86.29% |
| *Bacillus weihenstephanensis* KBAB4 | BcerKBAB4_2118 | L-lysine 2,3-aminomutase | TTGGCATAATTATTGCT | -38 | 90.30% |

| Strains | Gene ID | Annotations | -12/-24 sequence | Position* | Identity |
|---|---|---|---|---|---|
| *Bacillus thuringiensis* serovar *kurstaki* HD73 | HD73_3140 | hypothetical protein | TTGGCATGATTTTTGCA | -41 | |
| *Bacillus thuringiensis* BMB171 | BMB171_C2540 | hypothetical protein | TTGGCATGATTTTTGCT | -40 | 98.90% |
| *Bacillus thuringiensis* 97-27 | BT9727_2597 | hypothetical protein | TTGGCATATTTTTTGCA | -40 | 89.38% |
| *Bacillus cereus* ATCC 14579 | BC2838 | hypothetical protein | TTGGCATGATTTTTGCT | 11 | 83.33% |
| *Bacillus cereus* ATCC 10987 | BCE_2866 | hypothetical protein | TTGGCATAGTTTTTGCA | -41 | 89.01% |
| *Bacillus cereus* E33L | BCZK2562 | hypothetical protein | TTGGCATAGTTTTTGCA | -41 | 89.01% |
| *Bacillus cereus* FRI-35 | BCK_20685 | hypothetical protein | TTGGCATAGTTTTTGCA | -40 | 87.80% |
| *Bacillus anthracis* str. Ames | — | — | — | — | — |
| *Bacillus anthracis* A0248 | — | — | — | — | — |
| *Bacillus anthracis* CDC 684 | BAMEG_1759 | hypothetical protein | TTGGCATATTTTTTGCA | -168 | 48.35% |
| *Bacillus mycoides* DSM 2048 | — | — | — | — | — |
| *Bacillus pseudomycoides* DSM 12442 | — | — | — | — | — |
| *Bacillus weihenstephanensis* KBAB4 | BcerKBAB4_2640 | hypothetical protein | TTGGCATAGTTTTTGCA | -39 | 84.98% |

| Strains | Gene ID | Annotations | -12/-24 sequence | Position* | Identity |
|---|---|---|---|---|---|
| *Bacillus thuringiensis* serovar *kurstaki* HD73 | HD73_3142 | Sarcosine oxidase, beta subunit | TTGGCACGTCAATTGCA | -41 | |
| *Bacillus thuringiensis* BMB171 | BMB171_C2538 | Sarcosine oxidase, beta subunit | TTGGCACGTCAATTGCA | -41 | 97.64% |
| *Bacillus thuringiensis* 97-27 | BT9727_2595 | Sarcosine oxidase, beta subunit | TTGGCACGTCAATTGCA | -41 | 88.01% |
| *Bacillus cereus* ATCC 14579 | BC2836 | Sarcosine oxidase, beta subunit | TTGGCACGTCAATTGCA | -41 | 97.19% |
| *Bacillus cereus* ATCC 10987 | BCE_2864 | Sarcosine oxidase, beta subunit | TTGGCACGTCAATTGCA | -41 | 84.10% |
| *Bacillus cereus* E33L | BCZK2560 | Sarcosine oxidase, beta subunit | TTGGCACGTCAATTGCA | -41 | 88.27% |
| *Bacillus cereus* FRI-35 | BCK_20695 | Glycine oxidase | TTGGCACGTCAATTGCA | -40 | 95.10% |
| *Bacillus anthracis* str. Ames | BA_2835 | Sarcosine oxidase, beta subunit | TTGGCACGTCAATTGCA | -41 | 87.93% |
| *Bacillus anthracis* A0248 | BAA_2897 | Sarcosine oxidase, beta subunit | TTGGCACGTCAATTGCA | -41 | 87.93% |
| *Bacillus anthracis* CDC 684 | BAMEG_1761 | Sarcosine oxidase, beta subunit | TTGGCACGTCAATTGCA | -41 | 87.93% |
| *Bacillus mycoides* DSM 2048 | bmyco0001_25390 | Sarcosine oxidase, beta subunit | TTGGCACACCAATTGCA | -43 | 88.35% |
| *Bacillus pseudomycoides* DSM 12442 | bpmyx0001_23940 | Sarcosine oxidase, beta subunit | TTGGCATACCAATTGCA | -225 | 71.26% |
| *Bacillus weihenstephanensis* KBAB4 | BcerKBAB4_2638 | Sarcosine oxidase, beta subunit | TTGGCACACCAATTGCA | -43 | 88.52% |

| Strains | Gene ID | Annotations | -12/-24 sequence | Position* | Identity |
|---|---|---|---|---|---|
| *Bacillus thuringiensis* serovar *kurstaki* HD73 | HD73_4161 | proline dipeptidase | TTGGCACGCTATTTGCT | -32 | |
| *Bacillus thuringiensis* BMB171 | BMB171_C3542 | Xaa-Pro dipeptidase | CTGGCACAATTCTTGCT | -28 | 88.33% |
| *Bacillus thuringiensis* 97-27 | BT9727_3617 | proline dipeptidase | TTGGCACACTATTTGCT | -32 | 91.22% |
| *Bacillus cereus* ATCC 14579 | BC3875 | Xaa-Pro dipeptidase | CTGGCACAATTCTTGCT | -28 | 87.68% |
| *Bacillus cereus* ATCC 10987 | BCE_3921 | proline dipeptidase | TTGGCACAATTTTTGCT | -29 | 91.69% |
| *Bacillus cereus* E33L | BCZK3635 | proline dipeptidase | TTGGCACACTATTTGCT | -32 | 91.50% |
| *Bacillus cereus* FRI-35 | BCK_15855 | proline dipeptidase | TTGGCACAATTTTTGCT | -29 | 93.50% |
| *Bacillus anthracis* str. Ames | BA_4014 | proline dipeptidase | TTGGCACACTATTTGCT | -32 | 91.32% |
| *Bacillus anthracis* A0248 | BAA_4038 | Xaa-Pro dipeptidase | TTGGCACACTATTTGCT | -32 | 91.32% |
| *Bacillus anthracis* CDC 684 | BAMEG_0616 | Xaa-Pro dipeptidase | TTGGCACACTATTTGCT | -32 | 91.32% |
| *Bacillus mycoides* DSM 2048 | bmyco0001_34980 | proline dipeptidase | TTGGCACGCTATTTGCT | -33 | 93.46% |
| *Bacillus pseudomycoides* DSM 12442 | bpmyx0001_33440 | proline dipeptidase | TTGGCATAATTATTGCT | -31 | 75.63% |
| *Bacillus weihenstephanensis* KBAB4 | BcerKBAB4_3699 | peptidase M24 | TTGGCACGCTATTTGCT | -33 | 92.81% |

| Strains | Gene ID | Annotations | -12/-24 sequence | Position* | Identity |
|---|---|---|---|---|---|
| *Bacillus thuringiensis* serovar *kurstaki* HD73 | HD73_3213 | Acetoin:2,6-dichlorophenolindophenol oxidoreductase subunit alpha | TTGGCACGGTACTTGCA | -37 | |
| *Bacillus thuringiensis* BMB171 | BMB171_C2486 | Acetoin dehydrogenase E1 component subunit alpha | TTGGCACGGTACTTGCA | -37 | 63.16% |
| *Bacillus thuringiensis* 97-27 | BT9727_2539 | Acetoin dehydrogenase (TPP-dependent) E1 component subunit alpha | TTGGCACGGTACTTGCG | -37 | 60.86% |
| *Bacillus cereus* ATCC 14579 | BC2779 | Acetoin dehydrogenase E1 component alpha-subunit | TTGGCACGGTACTTGCA | -37 | 62.66% |
| *Bacillus cereus* ATCC 10987 | BCE_2804 | Acetoin dehydrogenase E1 component subunit alpha | TTGGCACAGTACTTGCA | -37 | 60.66% |
| *Bacillus cereus* E33L | BCZK2505 | Acetoin dehydrogenase (TPP-dependent) E1 component subunit alpha | TTGGCACGGTACTTGCA | -37 | 60.96% |
| *Bacillus cereus* FRI-35 | BCK_21020 | TPP-dependent acetoin dehydrogenase E1 subunit alpha | TTGGCACGGTACTTGCT | -37 | 98.30% |
| *Bacillus anthracis* str. Ames | BA_2776 | Acetoin dehydrogenase E1 component alpha-subunit | TTGGCACAGTACTTGCA | -37 | 61.56% |
| *Bacillus anthracis* A0248 | BAA_2839 | Acetoin dehydrogenase E1 component subunit alpha | TTGGCACAGTACTTGCA | -37 | 61.56% |
| *Bacillus anthracis* CDC 684 | BAMEG_1820 | Acetoin dehydrogenase E1 component subunit alpha | TTGGCACAGTACTTGCA | -37 | 61.56% |
| *Bacillus mycoides* DSM 2048 | bmyco0001_24790 | Pyruvate dehydrogenase, TPP-dependent E1 component alpha-subunit | TTGGCACAGTACTTGCA | -10 | 59.26% |
| *Bacillus pseudomycoides* DSM 12442 | — | — | — | — | — |
| *Bacillus weihenstephanensis* KBAB4 | BcerKBAB4_2578 | pyruvate dehydrogenase (acetyl-transferring) | TTGGCACAGTACTTGCA | -37 | 60.86% |

| Strains | Gene ID | Annotations | -12/-24 sequence | Position* | Identity |
|---|---|---|---|---|---|
| *Bacillus thuringiensis* serovar *kurstaki* HD73 | HD73_4468 | phosphate butyryltransferase | TTGGCACGGTATTTGCT | -44 | |
| *Bacillus thuringiensis* BMB171 | BMB171_C3826 | phosphate butyryltransferase | TTGGCACGGTATTTGCT | -44 | 98.56% |
| *Bacillus thuringiensis* 97-27 | BT9727_3909 | phosphate butyryltransferase | TTGGCACGGTATTTGCT | -44 | 95.33% |
| *Bacillus cereus* ATCC 14579 | BC4163 | phosphate butyryltransferase | TTGGCACGGTATTTGCT | -44 | 98.67% |
| *Bacillus cereus* ATCC 10987 | BCE_4238 | phosphate butyryltransferase | TTGGCACGGTATTTGCT | -44 | 94.67% |
| *Bacillus cereus* E33L | BCZK3918 | phosphate butyryltransferase | TTGGCACGGTATTTGCT | -44 | 95.11% |
| *Bacillus cereus* FRI-35 | BCK_14350 | phosphate butyryltransferase | TTGGCACGGTATTTGCT | -44 | 96.70% |
| *Bacillus anthracis* str. Ames | BA_4388 | phosphate butyryltransferase | TTGGCACGGTATTTGCT | -44 | 95.11% |
| *Bacillus anthracis* A0248 | BAA_4407 | phosphate butyryltransferase | TTGGCACGGTATTTGCT | -44 | 95.11% |
| *Bacillus anthracis* CDC 684 | BAMEG_4425 | phosphate butyryltransferase | TTGGCACGGTATTTGCT | -44 | 95.00% |
| *Bacillus mycoides* DSM 2048 | bmyco0001_37910 | phosphate butyryltransferase | TTGGCACGGTATTTGCT | -44 | 93.44% |
| *Bacillus pseudomycoides* DSM 12442 | bpmyx0001_36480 | phosphate butyryltransferase | TTGGCACGATATTTGCT | -44 | 89.89% |
| *Bacillus weihenstephanensis* KBAB4 | BcerKBAB4_4007 | phosphate butyryltransferase | TTGGCACGGTATTTGCT | -44 | 93.22% |

| Strains | Gene ID | Annotations | -12/-24 sequence | Position* | Identity |
|---|---|---|---|---|---|
| *Bacillus thuringiensis* serovar *kurstaki* HD73 | HD73_5327 | NADPH dehydrogenase, quinone | TTGGCATATATGCTGCA | -613 | |
| *Bacillus thuringiensis* BMB171 | BMB171_C4569 | NADPH dehydrogenase, quinone | TTGGCATTGGTATTGTT | -172 | 97.42% |
| *Bacillus thuringiensis* 97-27 | BT9727_4666 | NADPH dehydrogenase, quinone | GTGGATTAGATATTCCA | -363 | 94.16% |
| *Bacillus cereus* ATCC 14579 | BC4958 | NADPH dehydrogenase, quinone | TTGGCATTGGTATTGTT | -172 | 97.25% |
| *Bacillus cereus* ATCC 10987 | BCE_5095 | NADPH dehydrogenase, quinone | TCGGCATATATGCCGCA | -602 | 94.16% |
| *Bacillus cereus* E33L | BCZK4683 | NADPH dehydrogenase, quinone | TTGGCTAGAGTGCT | -232 | 94.67% |
| *Bacillus cereus* FRI-35 | BCK_10480 | NADPH dehydrogenase, quinone | TCGGCATATATGCCGCA | -602 | 94.16% |
| *Bacillus anthracis* str. Ames | BA_5191 | NADPH dehydrogenase, quinone | ATGCTATATATACTCAT | -573 | 94.67% |
| *Bacillus anthracis* A0248 | BAA_5227 | NADPH dehydrogenase, quinone | ATGCTATATATACTCAT | -525 | 87.46% |
| *Bacillus anthracis* CDC 684 | BAMEG_5248 | NADPH dehydrogenase, quinone | ATGCTATATATACTCAT | -525 | 87.46% |
| *Bacillus mycoides* DSM 2048 | bmyco0001_45490 | NADPH dehydrogenase, quinone | TTTTTATATATTCGTGT | -42 | 91.58% |
| *Bacillus pseudomycoides* DSM 12442 | bpmyx0001_44150 | NADPH dehydrogenase, quinone | TAAATATATATTCTGCT | -205 | 84.62% |
| *Bacillus weihenstephanensis* KBAB4 | BcerKBAB4_4778 | NADPH dehydrogenase, quinone | TTGGCTCATCTGCT | -785 | 93.64% |

| **Strains** | **Gene ID** | **Annotations** | **-12/-24 sequence** | **Position*** | **Identity** |
|---|---|---|---|---|---|
| *Bacillus thuringiensis* serovar *kurstaki* HD73 | HD73_5614 | PTS system cellobiose-specific IIC component | TTGGCACGCTAATTGCA | -387 | |
| *Bacillus thuringiensis* BMB171 | BMB171_C4803 | PTS system, cellobiose-specific IIB component | TTGGCACGCTAATTGCA | -69 | 96.70% |
| *Bacillus thuringiensis* 97-27 | BT9727_4889 | PTS system, cellobiose-specific IIB component | TTGGCACGTTAATTGCA | -69 | 95.38% |
| *Bacillus cereus* ATCC 14579 | BC5217 | PTS system, cellobiose-specific IIB component | ATGGTGCACTCCTTGCC | -36 | 63.70% |
| *Bacillus cereus* ATCC 10987 | BCE_5321 | PTS system, cellobiose-specific IIB component | TTGGCACGCTAATTGCA | -69 | 95.38% |
| *Bacillus cereus* E33L | BCZK4904 | PTS system, lichenan-specific IIB component | TTGGCACGCTAATTGCA | -68 | 96.04% |
| *Bacillus cereus* FRI-35 | BCK_09340 | PTS system, cellobiose-specific IIB component | TTGGCACGCTAATTGCA | -69 | 96.37% |
| *Bacillus anthracis* str. Ames | BA_5444 | PTS system, lichenan-specific IIB component | TTGGCACGCTAATTGCA | -69 | 96.37% |
| *Bacillus anthracis* A0248 | BAA_5472 | PTS system, cellobiose-specific IIB component | TTGGCACGCTAATTGCA | -69 | 96.37% |
| *Bacillus anthracis* CDC 684 | BAMEG_5494 | PTS system, cellobiose-specific IIB component | TTGGCACGCTAATTGCA | -69 | 96.37% |
| *Bacillus mycoides* DSM 2048 | bmyco0001_47800 | PTS system, lichenan-specific IIB component | TTGGCACGCTAATTGCA | -69 | 95.05% |
| *Bacillus pseudomycoides* DSM 12442 | bpmyx0001_46410 | PTS system, lichenan-specific IIB component | TTGGCACGGAAGTTGCA | -66 | 91.42% |
| *Bacillus weihenstephanensis* KBAB4 | BcerKBAB4_5004 | PTS system, cellobiose-specific IIB component | TTGGCACGCTAATTGCA | -68 | 95.05% |

| **Strains** | **Gene ID** | **Annotations** | **-12/-24 sequence** | **Position*** | **Identity** |
|---|---|---|---|---|---|
| *Bacillus thuringiensis* serovar *kurstaki* HD73 | HD73_0179 | pyrroline-5-carboxylate reductase | TTGGTATGACAAAAGCA | -289 | |
| *Bacillus thuringiensis* BMB171 | BMB171_C0181 | pyrroline-5-carboxylate reductase | TTGGTATGACAAAAGCA | -289 | 97.4% |
| *Bacillus thuringiensis* 97-27 | BT9727_0185 | pyrroline-5-carboxylate reductase | TTGTGTAATAAAAATA | -374 | 93.3% |
| *Bacillus cereus* ATCC 14579 | BC0218 | pyrroline-5-carboxylate reductase | TTGGTATGACAAAAGCA | -289 | 98.1% |
| *Bacillus cereus* ATCC 10987 | BCE_0217 | pyrroline-5-carboxylate reductase | TAGAAACGACTAAAAAA | -205 | 94.4% |
| *Bacillus cereus* E33L | BCZK0188 | pyrroline-5-carboxylate reductase | CTTGTGTAATAAAAATA | -373 | 93.6% |
| *Bacillus cereus* FRI-35 | BCK_06935 | pyrroline-5-carboxylate reductase | CTTGTGTAATAAAAATA | -372 | 95.1% |
| *Bacillus anthracis* str. Ames | BA_0197 | pyrroline-5-carboxylate reductase | CTTGTGTAATAAAAATA | -372 | 93.5% |
| *Bacillus anthracis* A0248 | BAA_0230 | oxidoreductase, aldo/keto reductase family | TTGGATTATGCCAAATACA | -434 | 92% |
| *Bacillus anthracis* CDC 684 | BAMEG_0231 | pyrroline-5-carboxylate reductase | CTTGTGTAATAAAAATA | -372 | 93.5% |
| *Bacillus mycoides* DSM 2048 | — | — | — | — | — |
| *Bacillus pseudomycoides* DSM 12442 | — | — | — | — | — |
| *Bacillus weihenstephanensis* KBAB4 | BcerKBAB4_0182 | pyrroline-5-carboxylate reductase | CTTGTGTAGTGAAAATA | -372 | 83% |

| Strains | Gene ID | Annotations | -12/-24 sequence | Position* | Identity |
|---|---|---|---|---|---|
| *Bacillus thuringiensis* serovar *kurstaki* HD73 | HD73_1649 | diaminopimelate decarboxylase | TTGGAGATGTTGATGCG | -169 | |
| *Bacillus thuringiensis* BMB171 | BMB171_C1256 | diaminopimelate decarboxylase | TTGGAGATGTTGATGCG | -169 | 98.9% |
| *Bacillus thuringiensis* 97-27 | BT9727_1302 | diaminopimelate decarboxylase | TTGGAGATGTTGATGCG | -169 | 92% |
| *Bacillus cereus* ATCC 14579 | BC1419 | diaminopimelate decarboxylase | TTGGAGATGTTGATGCG | -169 | 98.4% |
| *Bacillus cereus* ATCC 10987 | BCE_1542 | diaminopimelate decarboxylase | TTGGAGATGTTGATGCG | -169 | 95.9% |
| *Bacillus cereus* E33L | BCZK1303 | diaminopimelate decarboxylase | TTGGAGATGTTGATGCG | -169 | 95.7% |
| *Bacillus cereus* FRI-35 | BCK_01280 | diaminopimelate decarboxylase | TTGGAGATGTTGATGCG | -169 | 95.9% |
| *Bacillus anthracis* str. Ames | BA_1438 | diaminopimelate decarboxylase | TTGGAGATGTTGATGCG | -169 | 95.9% |
| *Bacillus anthracis* A0248 | BAA_1507 | diaminopimelate decarboxylase | TTGGAGATGTTGATGCG | -169 | 95.9% |
| *Bacillus anthracis* CDC 684 | BAMEG_3155 | diaminopimelate decarboxylase | TTGGAGATGTTGATGCG | -169 | 95.9% |
| *Bacillus mycoides* DSM 2048 | — | — | — | — | — |
| *Bacillus pseudomycoides* DSM 12442 | — | — | — | — | — |
| *Bacillus weihenstephanensis* KBAB4 | BcerKBAB4_1342 | diaminopimelate decarboxylase | TTGGAGATGTTGATGCG | -169 | 94.3% |

| Strains | Gene ID | Annotations | -12/-24 sequence | Position* | Identity |
|---|---|---|---|---|---|
| *Bacillus thuringiensis* serovar *kurstaki* HD73 | HD73_2025 | branched-chain amino acid aminotransferase | TCGGAGCATCGCTTGCG | -590 | |
| *Bacillus thuringiensis* BMB171 | BMB171_C1642 | branched-chain amino acid aminotransferase | TCGGAGCATCGCTTGCG | -591 | 98.11% |
| *Bacillus thuringiensis* 97-27 | BT9727_1690 | branched-chain amino acid aminotransferase | TCGGAGCATCGCTTGCG | -592 | 92.33% |
| *Bacillus cereus* ATCC 14579 | BC1776 | branched-chain amino acid aminotransferase | TCGGAGCATCGCTTGCG | -612 | 95.67% |
| *Bacillus cereus* ATCC 10987 | BCE_1933 | branched-chain amino acid aminotransferase | TCGGAGCATCGCTTGCG | -588 | 94.22% |
| *Bacillus cereus* E33L | BCZK1665 | branched-chain amino acid aminotransferase | TCGGAGCATCGCTTGCG | -592 | 92.00% |
| *Bacillus cereus* FRI-35 | BCK_25345 | branched-chain amino acid aminotransferase | TCGGAGCATCGCTTGCG | -588 | 94.33% |
| *Bacillus anthracis* str. Ames | BA_1849 | branched-chain amino acid aminotransferase | TCGGAGCATCGCTTGCG | -592 | 92.00% |
| *Bacillus anthracis* A0248 | BAA_1918 | branched-chain amino acid aminotransferase | TCGGAGCATCGCTTGCG | -592 | 92.00% |
| *Bacillus anthracis* CDC 684 | BAMEG_2743 | branched-chain amino acid aminotransferase | TCGGAGCATCGCTTGCG | -592 | 92.00% |
| *Bacillus mycoides* DSM 2048 | bmyco0001_16380 | branched-chain amino acid aminotransferase | TCGGAGCATCGCTTGCG | -611 | 89.22% |
| *Bacillus pseudomycoides* DSM 12442 | bpmyx0001_16220 | branched-chain amino acid aminotransferase | TTGGAGCATCGCTTGCG | -617 | 82.00% |
| *Bacillus weihenstephanensis* KBAB4 | BcerKBAB4_1706 | branched-chain amino acid aminotransferase | TCGGAGCATCGCTTACG | -590 | 90.67% |

| Strains | Gene ID | Annotations | -12/-24 sequence | Position* | Identity |
|---|---|---|---|---|---|
| *Bacillus thuringiensis* serovar *kurstaki* HD73 | HD73_4943 | Acetate-CoA ligase | ATGGCTTAGAAAGAGCG | -166 | |
| *Bacillus thuringiensis* BMB171 | BMB171_C4285 | Acetyl-CoA synthetase | ATGGCTTAGAAAGAGCG | -172 | 98.80% |
| *Bacillus thuringiensis* 97-27 | BT9727_4379 | Acetate-CoA ligase | ATGGCTTAGAAAGAGCG | -157 | 93.45% |
| *Bacillus cereus* ATCC 14579 | BC4645 | Acetyl-CoA synthetase | ATGGCTTAGAAAGAGCG | -172 | 98.36% |
| *Bacillus cereus* ATCC 10987 | BCE_4781 | Acetyl-CoA synthetase | ATGGCTTAGAAAGAGCG | -208 | 93.38% |
| *Bacillus cereus* E33L | BCZK4389 | Acetate-CoA ligase | ATGGCTTAGAAAGAGCG | -252 | 92.88% |
| *Bacillus cereus* FRI-35 | BCK_11970 | Acetate-CoA ligase | ATGGCTTAGAAAGAGCG | -190 | 94.33% |
| *Bacillus anthracis* str. Ames | BA_4896 | Acetyl-CoA synthetase | ATGGCTTAGAAAGAGCG | -270 | 92.19% |
| *Bacillus anthracis* A0248 | BAA_4907 | acetyl-CoA synthetase | ATGGCTTAGAAAGAGCG | -252 | 93.26% |
| *Bacillus anthracis* CDC 684 | BAMEG_4928 | acetyl-CoA synthetase | ATGGCTTAGAAAGAGCG | -252 | 93.26% |
| *Bacillus mycoides* DSM 2048 | bmyco0001_42590 | Acetate-CoA ligase | ATGGCTTAGAAAGAGCG | -194 | 89.67% |
| *Bacillus pseudomycoides* DSM 12442 | bpmyx0001_41090 | Acetate-CoA ligase | ATGGCTTAGAAGGAGCG | -125 | 82.99% |
| *Bacillus weihenstephanensis* KBAB4 | BcerKBAB4_4476 | acetyl-CoA synthetase | ATGGCTTAGAAAGAGCG | -194 | 89.92% |

*****Distance between the -12 region of the promoter relative to the initiation codon.

—: Not found in the special strain.
