# Supplementary material for: Identification of metabolism pathways directly regulated by sigma54 factor in Bacillus thuringiensis
Source: Front Microbiol. 2015 May 12;6:407. doi: 10.3389/fmicb.2015.00407 (PMC4428206; doi:10.3389/fmicb.2015.00407)
Supplement: Supplementary file 4 [file Table4.PDF]

Additional file 4. Strains and plasmids used in this study

Strain or plasmid	Relevant genotype and characteristics[a]	Reference or source
**Strains**		
HD73	*Bacillus thuringiensis* subsp. *Kurstaki* carrying the *cry1Ac* gene	Laboratory collection
HD(Δ*sigL*)	Bt HD73 *sigL* gene deletion mutant; Kan^R	Zhu, et al., 210
HD(Δ*gabR*)	Bt HD73 *HD73_0367* gene deletion mutant; Kan^R	Zhu, et al., 210
HD(Δ*rocR*)	Bt HD73 *HD73_0559* gene deletion mutant; Kan^R	This study
HD(Δ*prdR*)	Bt HD73 *HD73_1069* gene deletion mutant; Kan^R	This study
HD(Δ*kamR*)	Bt HD73 *HD73_2539* gene deletion mutant; Kan^R	Zhang, et al., 2014
HD(Δ*soxR*)	Bt HD73 *HD73_3141* gene deletion mutant;	This study
HD(Δ*acoR*)	Bt HD73 *HD73_3228* gene deletion mutant; Kan^R	This study
HD(Δ*bkdR*)	Bt HD73 *HD73_4469* gene deletion mutant; Kan^R	This study
HD(Δ*levR*)	Bt HD73 *HD73_5607* gene deletion mutant; Kan^R	This study
HD(P*0560*)	Bt HD73 carrying pHT-P0560 plasmid; Em^R	This study
Δ*sigL*(P*0560*)	HD(Δ*sigL*) carrying pHT-P0560 plasmid; Em^R; Kan^R	This study
Δ*rocR*(P*0560*)	HD(Δ*rocR*) carrying pHT-P0560 plasmid; Em^R; Kan^R	This study
HD(P*1024*)	Bt HD73 carrying pHT-P1024 plasmid; Em^R	This study
Δ*sigL*(P*1024*)	HD(Δ*sigL*) carrying pHT-P1024 plasmid; Em^R; Kan^R	This study
Δ*prdR*(P*1024*)	HD(Δ*prdR*) carrying pHT-P1024 plasmid; Em^R; Kan^R	This study
HD(P*1070*)	Bt HD73 carrying pHT-P1070 plasmid; Em^R	This study
Δ*sigL*(P*1070*)	HD(Δ*sigL*) carrying pHT-P1070 plasmid; Em^R; Kan^R	This study
Δ*prdR*(P*1070*)	HD(Δ*prdR*) carrying pHT-P1070 plasmid; Em^R; Kan^R	This study
HD(P*2699*)	Bt HD73 carrying pHT-P2699 plasmid; Em^R	This study
Δ*sigL*(P*2699*)	HD(Δ*sigL*) carrying pHT-P2699 plasmid; Em^R; Kan^R	This study
HD(P*2953*)	Bt HD73 carrying pHT-P2953 plasmid; Em^R	This study
Δ*sigL*(P*2953*)	HD(Δ*sigL*) carrying pHT-P2953 plasmid; Em^R; Kan^R	This study
HD(P*3140*)	Bt HD73 carrying pHT-P3140 plasmid; Em^R	This study
Δ*sigL*(P*3140*)	HD(Δ*sigL*) carrying pHT-P3140 plasmid; Em^R; Kan^R	This study
Δ*soxR*(P*3140*)	HD(Δ*sigL*) carrying pHT-P3140 plasmid; Em^R	This study
HD(P*3142*)	Bt HD73 carrying pHT-P3142 plasmid; Em^R	This study
Δ*sigL*(P*3142*)	HD(Δ*sigL*) carrying pHT-P3142 plasmid; Em^R; Kan^R	This study
Δ*soxR*(P*3142*)	HD(Δ*soxR*) carrying pHT-P312 plasmid; Em^R	This study
HD(P*3213*)	Bt HD73 carrying pHT-P3213 plasmid; Em^R	This study
Δ*sigL*(P*3213*)	HD(Δ*sigL*) carrying pHT-P3213 plasmid; Em^R; Kan^R	This study
Δ*acoR*(P*3213*)	HD(Δ*acoR*) carrying pHT-P3213 plasmid; Em^R; Kan^R	This study
HD(P*4161*)	Bt HD73 carrying pHT-P4161 plasmid; Em^R	This study
Δ*sigL*(P*4161*)	HD(Δ*sigL*) carrying pHT-P4161 plasmid; Em^R; Kan^R	This study
HD(P*4468*)	Bt HD73 carrying pHT-P4468 plasmid; Em^R	This study
Δ*sigL*(P*4468*)	HD(Δ*sigL*) carrying pHT-P4468 plasmid; Em^R; Kan^R	This study
Δ*bkdR*(P*4468*)	HD(Δ*bkdR*) carrying pHT-P4468 plasmid; Em^R; Kan^R	This study

HD(P*5327*)	Bt HD73 carrying pHT-P5327 plasmid; Em^R	This study
Δ*sigL*(P*5327*)	HD(Δ*sigL*) carrying pHT-P5327 plasmid; Em^R; Kan^R	This study
HD(P*5614*)	Bt HD73 carrying pHT-P5614 plasmid; Em^R	This study
Δ*sigL*(P*5614*)	HD(Δ*sigL*) carrying pHT-P5614 plasmid; Em^R; Kan^R	This study
Δ*levR*(P*5614*)	HD(Δ*levR*) carrying pHT-P5614 plasmid; Em^R; Kan^R	This study
HD(P*0035*)	Bt HD73 carrying pHT-P0035 plasmid; Em^R	This study
Δ*sigL*(P*0035*)	HD(Δ*sigL*) carrying pHT-P0035 plasmid; Em^R; Kan^R	This study
HD(P*0179*)	Bt HD73 carrying pHT-P0179 plasmid; Em^R	This study
Δ*sigL*(P*0179*)	HD(Δ*sigL*) carrying pHT-P0179 plasmid; Em^R; Kan^R	This study
HD(P*1649*)	Bt HD73 carrying pHT-P1649 plasmid; Em^R	This study
Δ*sigL*(P*1649*)	HD(Δ*sigL*) carrying pHT-P1649 plasmid; Em^R; Kan^R	This study
HD(P*1772*)	Bt HD73 carrying pHT-P1772 plasmid; Em^R	This study
Δ*sigL*(P*1772*)	HD(Δ*sigL*) carrying pHT-P1772 plasmid; Em^R; Kan^R	This study
HD(P*2025*)	Bt HD73 carrying pHT-P2025 plasmid; Em^R	This study
Δ*sigL*(P*2025*)	HD(Δ*sigL*) carrying pHT-P2025 plasmid; Em^R; Kan^R	This study
HD(P*4943*)	Bt HD73 carrying pHT-P4943 plasmid; Em^R	This study
Δ*sigL*(P*4943*)	HD(Δ*sigL*) carrying pHT-P4943 plasmid; Em^R; Kan^R	This study
HD(P*4960*)	Bt HD73 carrying pHT-P4960 plasmid; Em^R	This study
Δ*sigL*(P*4960*)	HD(Δ*sigL*) carrying pHT-P4960 plasmid; Em^R; Kan^R	This study
E. coli TG1	Δ(*lac-proAB*) *supE thi hsd*-5 (*F' traD36* $proA^+$ $proB^+$ $lacI^q$ *lac*ZΔM15), general purpose cloning host	Laboratory collection
E. coli ET12567	F^- *dam-13*::Tn*9 dcm-6 hsdM hsdR recF143 zjj-202*: :Tn*10 galK2 galT22 ara14 pacY1 xyl-5 leuB6 thi-1*, for generation of unmethylated DNA	Laboratory collection
**Plasmids**		
pHT304-18Z	Promoterless *lacZ* vector, Em^R, Ap^R	Agaisse and Lereclus, 1994
pMAD	Ap^R, Em^R shuttle vector, thermosensitive origin of replication	Arnaud, et al., 2004
pHT-P0560	pHT304-18Z carrying promoter upstream from *hd73_0560*	This study
pHT-P1024	pHT304-18Z carrying promoter upstream from *hd73_1024*	This study
pHT-P1070	pHT304-18Z carrying promoter upstream from *hd73_1070*	This study
pHT-P2699	pHT304-18Z carrying promoter upstream from *hd73_2699*	This study
pHT-P2953	pHT304-18Z carrying promoter upstream from *hd73_2953*	This study
pHT-P3140	pHT304-18Z carrying promoter upstream from *hd73_3140*	This study
pHT-P3142	pHT304-18Z carrying promoter upstream from *hd73_3142*	This study
pHT-P3213	pHT304-18Z carrying promoter upstream from *hd73_3213*	This study
pHT-P4161	pHT304-18Z carrying promoter upstream from *hd73_4161*	This study
pHT-P4468	pHT304-18Z carrying promoter upstream from *hd73_4468*	This study
pHT-P5327	pHT304-18Z carrying promoter upstream from *hd73_5327*	This study
pHT-P5614	pHT304-18Z carrying promoter upstream from *hd73_5614*	This study
pHT-P0035	pHT304-18Z carrying promoter upstream from *hd73_0035*	This study
pHT-P0179	pHT304-18Z carrying promoter upstream from *hd73_0179*	This study
pHT-P1649	pHT304-18Z carrying promoter upstream from *hd73_1649*	This study

pHT-P1772	pHT304-18Z carrying promoter upstream from *hd73_1772*	This study
pHT-P2025	pHT304-18Z carrying promoter upstream from *hd73_2025*	This study
pHT-P4943	pHT304-18Z carrying promoter upstream from *hd73_4943*	This study
pHT-P4960	pHT304-18Z carrying promoter upstream from *hd73_4960*	This study
pMADΔrocR	pMAD with *hd73_0559* deletion fragment	This study
pMADΔprdR	pMAD with *hd73_1069* deletion fragment	This study
pMADΔsoxR	pMAD with *hd73_3143* deletion fragment	This study
pMADΔacoR	pMAD with *hd73_3228* deletion fragment	This study
pMADΔbkdR	pMAD with *hd73_4469* deletion fragment	This study
pMADΔlevR	pMAD with *hd73_5607* deletion fragment	This study

Ap, ampicillin; Em, erythromycin; Kan, kanamycine
