# Supplementary material for: Identification of metabolism pathways directly regulated by sigma54 factor in Bacillus thuringiensis
Source: Front Microbiol. 2015 May 12;6:407. doi: 10.3389/fmicb.2015.00407 (PMC4428206; doi:10.3389/fmicb.2015.00407)
Supplement: Supplementary file 5 [file Table5.PDF]

Additional file 5. Primer sequences used in this study

oligonucleotides	sequence (5' → 3')[a]
rocR-AF	CGGGATCCTTTATCAGCAATGGCTTGTC
rocR-AR	CACCTCAAATGGTTCGCTGTCTGAGCGTTCCATTGATTC
rocR-BF	GAGCGCCTACGAGGAATTTGCACACAATAGAAGAAATGG
rocR-BR	CGGAATTCAGGACAATTAGACGCTGAAG
rocR-kmF	GAATCAATGGAACGCTCAGACAGCGAACCATTTGAGGTG
rocR-kmR	CCATTTCTTCTATTGTGTGCAAATTCCTCGTAGGCGCTC
prdR-AF	CGGGATCCACCTGCTCGTTGAAGTGAAC
prdR-AR	CACCTCAAATGGTTCGCTGGTAGGGCTTTTCGTGTTTCT
prdR-BF	GAGCGCCTACGAGGAATTTGAGGCACAGGTTAGACACCG
prdR-BR	CGGAATTCCGATAAATAAGACAGAAGCGAG
prdR-kmF	AGAAACACGAAAAGCCCTACCAGCGAACCATTTGAGGTG
prdR-kmR	CGGTGTCTAACCTGTGCCTCAAATTCCTCGTAGGCGCTC
acoR-AF	CGGGATCCATTAGAAGTGACGAGAGGCATA
acoR-AR	CACCTCAAATGGTTCGCTGTGCCACGATTCTGAAATTCT
acoR-BF	GAGCGCCTACGAGGAATTTACAGCGTGATAGCATGATTG
acoR-BR	CGGAATTCCAGCACACTTTTTCTTTATTCAG
acoR-kmF	AGAATTTCAGAATCGTGGCACAGCGAACCATTTGAGGTG
acoR-kmR	CAATCATGCTATCACGCTGTAAATTCCTCGTAGGCGCTC
bkdR-AF	CGGGATCCATTACTGATTACCCTGAAACGG
bkdR-AR	CACCTCAAATGGTTCGCTGTTGTAGTCCTTTTGCCAACG
bkdR-BF	GAGCGCCTACGAGGAATTTATTTGGAGAGATTTGATGGG
bkdR-BR	CGGAATTCTTCCCCTTCATAAGCACATC
bkdR-kmF	CGTTGGCAAAAGGACTACAACAGCGAACCATTTGAGGTG
bkdR-kmR	CCCATCAAATCTCTCCAAATAAATTCCTCGTAGGCGCTC
levR-AF	CGGGATCCCCTTTACACTGGTGACCCTTC
levR-AR	CACCTCAAATGGTTCGCTGTAGCGAGTTCTGATGCGGTT
levR-BF	GAGCGCCTACGAGGAATTTCGTTGCAACCAATTGAAAAC
levR-BR	CGGAATTCGAATGTAGAGGATTTTGAGGGA
levR-kmF	AACCGCATCAGAACTCGCTACAGCGAACCATTTGAGGTG
levR-kmR	GTTTTCAATTGGTTGCAACGAAATTCCTCGTAGGCGCTC
soxR-a	CGGGATCCACCGCCTAATAAATCATCAGCAAA
soxR-b	CGGAATTCTATTTACTGTCATGCGGCATTCGG
soxR-c	GGAGGACATTATGGAGTGTAACAAGGGCTACT
socR-d	TAGCCCTTGTTACACTCCATAATGTCCTCCTG
P0560-F	CCAAGCTTCGTCTGCTTTAAGTGACTAATTTC
P0560-R	CGGGATCC ATTCTCCCCTCTAGAGATTTCG
P1024-F	CCAAGCTTGATCCATGAAGCAGGATTTGA
P1024-R	CGGGATCC TTCCATCTCT CCCCTATATGTT
P1070-F	CCAAGCTTAGAGCACCTCTTTAGTTG
P1070-R	CGGGATCCTTTCTCTCACCTCATAA
P2699-F	CCAAGCTTCCATCTCCTAACGTATG

P2699-R	CGGGATCCCTACTTTCCCCCTGTATG
P2953-F	CCAAGCTTTTATAAAAAATGTTGGC
P2953-R	CGGGATCCACCATTGCCCCCTAACAA
PsoxC-F	AACTGCAGCCGCCTAATAAATCATCAGC
PsoxC-R	CGGGATCCGAACAGTGTCCCCGATATTAC
PsoxB-F	AACTGCAGCCATCTGTCCAAGTTCGAA
PsoxB-R	CGGGATCCACCCTATTATTCCACCACC
P3213-F	CCAAGCTTAAGCTTTCTGGAAACCGAATG
P3213-R	CGGGATCCGTTCAGCCCTCCTATTTTCTTG
P4161-F	AACTGCAGTATGTAGCAAGTGATGGTACA
P4161-R	CGGGATCCATTAATTCCC CCTATTCACAGT
P4468-F	CCAAGCTTGGAGAGTTTAGGGAGGATTTAT
P4468-R	CGGGATCCATTTTGTAATCAACCCTTTCCG
P5327-F	CCAAGCTTCTTCGCACCACTTCATGG
P5327-R	CGGGATCCTTTTCTTCCTCCTATTTGTG
P5614-F	CCAAGCTTAGAAGCACTAGAGGAATTAAAGG
P5614-R	CGGGATCCTTCGCTTCCCCCCTAATTAT TT
P0035-F	CCAAGCTTGACTTATTTTCCTCTGTCATT
P0035-R	CGGGATCCAATTTTTTTCCTCCTAAAT
P0179-F	AACTGCAGCTTATTCTTACTTCTTTC
P0179-R	CGGGATCCATTCTTTATT CCTTTCTAG
P1649-F	CCAAGCTTTAAAAAATTGTTTGACAAAT
P1649-R	CGGGATCCTTAAATTCCT CCCTTATTT
P1772-F	AACTGCAGCTGCAATTCTCCTTTGTTAAG
P1772-R	CGGGATCCAAACTACTCC TCCTTTATGC
P2025-F	AACTGCAGAGTTTCCTCATCTCCTCATAAAC
P2025-R	CGGGATCCCTCACATCGC TCCTCTTTTT TC
P4943-F	CCAAGCTTTAAAACCATAAATTACC
P4943-R	CGGGATCCAAAATATCCT CCCTTTTC
P4960-F	CCAAGCTTTAAGAGTGAATATAAGTT
P4960-R	CGGGATCCATCCTACATA CTCTCCTTT

[a] Restriction enzyme sites are underscored
